# A slippery molecular assembly allows water as a self-erasable security marker

**DOI:** 10.1038/srep09842

**Published:** 2015-05-05

**Authors:** Rajasekaran Thirumalai, Rahul Dev Mukhopadhyay, Vakayil K. Praveen, Ayyappanpillai Ajayaghosh

**Affiliations:** 1 Photosciences and Photonics Group, Chemical Sciences and Technology Division, CSIR-National Institute for Interdisciplinary Science and Technology (CSIR-NIIST), Trivandrum 695019, India; 2 Academy of Scientific and Innovative Research (AcSIR), CSIR-National Institute for Interdisciplinary Science and Technology (CSIR-NIIST), Trivandrum 695019, India

## Abstract

Protection of currency and valuable documents from counterfeit continues to be a challenge. While there are many embedded security features available for document safety, they are not immune to forgery. Fluorescence is a sensitive property, which responds to external stimuli such as solvent polarity, temperature or mechanical stress, however practical use in security applications is hampered due to several reasons. Therefore, a simple and specific stimuli responsive security feature that is difficult to duplicate is of great demand. Herein we report the design of a fluorescent molecular assembly on which water behaves as a self-erasable security marker for checking the authenticity of documents at point of care. The underlying principle involves the disciplined self-assembly of a tailor-made fluorescent molecule, which initially form a weak blue fluorescence (*λ*_em_ = 425 nm, *Φ*_f_ = 0.13) and changes to cyan emission (*λ*_em_ = 488 nm,*Φ*_f_ = 0.18) in contact with water due to a reversible molecular slipping motion. This simple chemical tool, based on the principles of molecular self-assembly and fluorescence modulation, allows creation of security labels and optically masked barcodes for multiple documents authentication.

Water is the gift of Nature to the mankind and is required for everything in life. Therefore, finding a new use for water is practically impossible without an out-of-box thinking^1^,^2^,^3^. One such innovative thinking is whether a drop of water can secure the authenticity of a valuable document/currency or whether water can be used as environmentally benign ink for writing. Thoughts in this direction have resulted in a few reports on the use of water as marker/ink for writing^3^,^4^. These reports describe specially coated papers on which water induces a visible colour change due to the structural changes of the coated chemical. A step ahead is the concept of invisible marker/ink that has significance in maintaining secrecy, which is possible through exploiting the phenomenon of fluorescence. Fluorescence is an extremely sensitive property of certain class of molecules and is visible only upon illuminating with a suitable source of light^5^. Many aromatic molecules exhibit fluorescence and are widely used in materials and biology related applications, particularly in imaging and displays.

Self-erasable, writing or printing is a novel concept of temporary communication, which allows the re-use of the writing medium for a certain period of time^6^,^7^,^8^,^9^. Self-erasable printing inks reduce the usage of papers for printing and save millions of trees, helping reduction of green gas emission. Even though Xerox company came up with such an idea, the technology could not be successfully launched^10^. Nevertheless, there are many other possibilities of using the concept of self-erasable writing, particularly on fluorescent medium^11^. For example, such an idea can be exploited for creating reusable security labels for the protection of valuable documents such as currency bills^12^. Every year, millions of different currencies are being counterfeited across the globe, which is considered as an act of economic terrorism^13^,^14^. These illegal currencies are being used for underworld activities and promotion of terrorism. Even though there are several security features for protection, which include the use of fluorescent inks, counterfeiting continues to threaten currency security^12^,^13^,^14^. Fluorescent inks that are currently used for security applications are easily available and hence fluorescent security markers can be easily faked for illegal activities. Therefore, design of new fluorescent materials for security applications that are difficult to duplicate remains to be a priority area of innovative research.

Molecular assemblies are an interesting class of soft materials, which exploit the possibilities of various weak noncovalent forces as a glue to hold them together^15^,^16^,^17^. In recent times, fluorescent molecular assemblies have been used for sensing^18^,^19^,^20^ and imaging^21^,^22^ applications, taking advantage of energy or electron transfer processes. When fluorescent molecules self-assemble to form supramolecular architectures of different size and shape they become powerful than the individual molecules^23^,^24^,^25^,^26^,^27^,^28^. Such molecular assemblies respond strongly to the surrounding medium and to various stimuli. The fluorescence intensity or colour variations resulted by the influence of an external force can trigger a temporary signal^26^,^27^. Such temporary signal in response to a stimulus can be used for the creation of security labels for the protection of documents from unauthorized duplication. Processes such as aggregation induced enhanced emission (AIEE)^24^,^28^ and mechanical stress induced fluorescence modulation^26^ that are the consequences of intermolecular electronic coupling at the excited states can be effectively utilized for sensing and security applications and hence are commonly used. However, there are several such systems known and are easy to duplicate. Therefore, it is necessary to have a completely new approach to have fluorescent molecules based security systems with controlled fluorescence modulation.

We demonstrate here how chemistry of a fluorescent molecular assembly and the sensitivity of its fluorescence to an external stimulus can be combined to secure the authenticity of documents using the idea of controlled molecular slipping with an external stimulus. This method takes advantage of the hydrophilic-hydrophobic balance and the hydrogen-bonding to control the strength of molecular exciton coupling through reversible sliding of molecules that changes emission colour in the presence and absence of water. In order to achieve this, we rely on the simple idea of slipping of objects on a watery surface. On a molecular level, this idea is conceived and implemented by the wetting of organized molecular assemblies with water and forcing them to slip or slide with the help of an in situ generated force with some control. We hypothesised that rigid fluorescent π-systems without any functional groups on the aromatic rings may be ideal candidates if one end of the molecule is connected with a bulky hydrophilic chain through a hydrogen bonding linkage. The force to induce the molecular sliding is generated by triggering a slight expansion (breathing) of the hydrophilic chains attached to the molecule upon contact with water molecules.

## Results

### Molecular design and self-assembly

**Figure 1:**
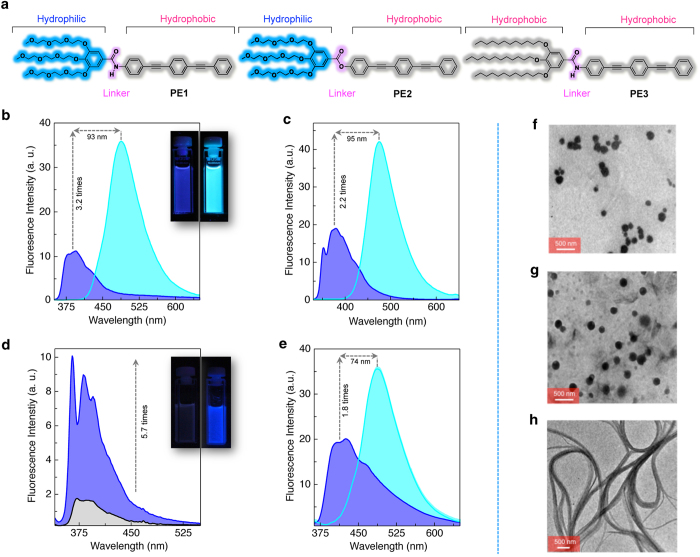
Controlling the emission and morphology of PE aggregates by molecular design. (**a**) Chemical structure of amphiphilic **PE1** and **PE2** and lipophilic **PE3** molecules used in this study. Emission spectra (*λ*_ex_ = 340 nm) of (**b**) **PE1** and (**c**) **PE2** in THF and THF-water mixture (1:9 v/v), *c* = 1 × 10^−5^ M. Inset of Fig. 1b shows photographs of **PE1** in THF (left) and THF-water mixture 1:9 v/v (right) under illumination at 365 nm. (**d**) Emission spectra (*λ*_ex_ = 340 nm) of **PE3** (*c* = 1 × 10^−4^ M) in chloroform and *n*-decane. Inset shows photographs of **PE3** in chloroform (left) and *n*-decane (right) under illumination at 365 nm. (**e**) Emission spectra (*λ*_ex_ = 340 nm) of **PE1** coated paper in the absence (blue) and presence of (cyan) water. TEM images of (**f**) **PE1** and (**g**) **PE2** aggregates prepared from THF-water mixture (1:9 v/v), *c* = 5 × 10^−5^ M displaying spherical particles and (**h**) **PE3** aggregates in *n*-decane (*c* = 5 × 10^−5^ M) showing 1-D fibre bundles.

For our studies, we designed three tailor-made molecular systems, **PE1**, **PE2** and **PE3** having the structures as shown in Fig. 1a. In these molecules, the triple bonded linear aromatic π-backbone (phenyleneethynylene, **PE**) is the fluorescent core, which is connected to a bulky end group through an amide or ester bond. The terminal bulky group is composed of flexible oxyethylene or alkyl chains. The amide linkage provides hydrogen bonds that control the molecular assembly whereas the ester linkage cannot provide such a control. These molecules were synthesized using multistep synthetic procedures using palladium-catalyzed Sonogashira–Hagihara cross coupling reactions (Supplementary Scheme S1 and S2). Chemical structures of these molecules were characterized by FT-IR, ^1^H and ^13^C NMR spectroscopy as well as by high-resolution mass spectrometry. These molecules were readily soluble in common organic solvents such as chloroform, toluene and tetrahydrofuran (THF). The UV/Vis absorption spectrum of **PE1** in THF (*c* = 1 × 10^−5^ M) at 25 °C showed an absorption band with *λ*_max_ at 336 nm (Supplementary Fig. S1). However, in THF-water mixture (1:9 v/v), the intensity of the absorption band is decreased with a small shift of the *λ*_max_ to 324 nm with a weak shoulder band at 388 nm (Supplementary Fig. S1).

The emission spectrum of **PE1** in THF (*c* = 1 × 10^−5^ M) exhibited a maximum at 395 nm when excited at 340 nm (Fig. 1b). The fluorescence quantum yield (*Φ*_f_) was estimated as 0.02 (±0.002) using quinine sulfate as a standard. Interestingly, in THF-water mixture (1:9 v/v), a significant red shift of the emission maximum (*λ*_em_ = 488 nm) with increased intensity (3.2 times) and quantum yield (*Φ*_f_ = 0.14 ± 0.05) were observed (Fig. 1b). This shift in the emission wavelength is obvious by the colour change from blue to intense cyan (Fig. 1b inset). This observation is an indication of aggregation induced enhanced emission (AIEE), which is a phenomenon associated with the aggregation of certain organic molecules^24^. Usually, AIEE is observed at the same wavelength position or with a slight shift with respect to the original emission of the molecule. Therefore, the large red shift of 93 nm (∆*ν* = 107527 cm^−1^) with enhanced fluorescent intensity^29^ observed in the case of the self-assembled **PE1** is unique and hence of great potential for applications. This enhanced emission at 488 nm occurs at above 60% water in THF (Supplementary Fig. S2). We then compared the fluorescence emission properties of **PE1** with those of **PE2** and **PE3**. The emission property of **PE2** in THF-water mixture was similar to that of **PE1** (Fig. 1c and Supplementary Fig. S3). The emission spectrum of **PE3** (*c* = 1 × 10^−4^ M) in chloroform was weak whereas the molecule exhibited enhanced fluorescence in *n*-decane indicati*n*g the AIEE behaviour (Fig. 1d and Supplementary Fig. S4). Thus, in practise, we could combine the AIEE property of **PE3** and the fluorescence shift of **PE2** in a single molecule of **PE1** by the rational choice of functional moieties. Fluorescence properties of **PE1**-**3** under different experimental conditions have been summarized in the Supplementary Table S1 and Supplementary Fig. S5 and Fig. S6.

The scanning electron microscopy (SEM) and transmission electron microscopy (TEM) analyses of the **PE1** in THF-water (1:9 v/v), drop cast on aluminium substrate and carbon coated copper grids (*c* = 5 × 10^−5^ M) exhibited spherical particles with average diameter of 100 nm (Fig. 1f and Supplementary Fig. S7). The spherical particle formation is further confirmed by dynamic light scattering (DLS) analysis (Supplementary Fig. S8) of the samples (*c* = 5 × 10^−5^ M) which showed average hydrodynamic radius (*R*_H_) of 122 nm. Fluorescence microscopy experiment also revealed the formation of fluorescent spherical particles (Supplementary Fig. S9). The spherical particles are stable enough without adding any stabilizers or surfactants. In order to know the stability of spherical aggregates, variable temperature emission study of **PE1** in 1:9 v/v THF-water mixture (*c* = 5 × 10^−5^ M) was carried out. The aggregates were heated from 20 to 80 °C at a heating rate of 1 °C per minute with constant stirring. Above 40 °C the aggregates start breaking and at 70 °C, the aggregates were completely dissociated which is clear from the plot of emission intensity monitored at 488 nm versus temperature (Supplementary Fig. S10). The morphological studies of **PE2** (Fig. 1g) also revealed the formation of spherical particles in THF-water mixture (1:9 v/v, *c* = 5 × 10^−5^ M). Interestingly, **PE3** with hydrophobic side chains in *n*-decane (*c* = 1 × 10^−4^ M) showed bundled fibrillar morphology, which is typical of 1-D lamellar assembly of the molecules (Fig. 1h).

### Response of the molecular assembly with water

**Figure 2:**
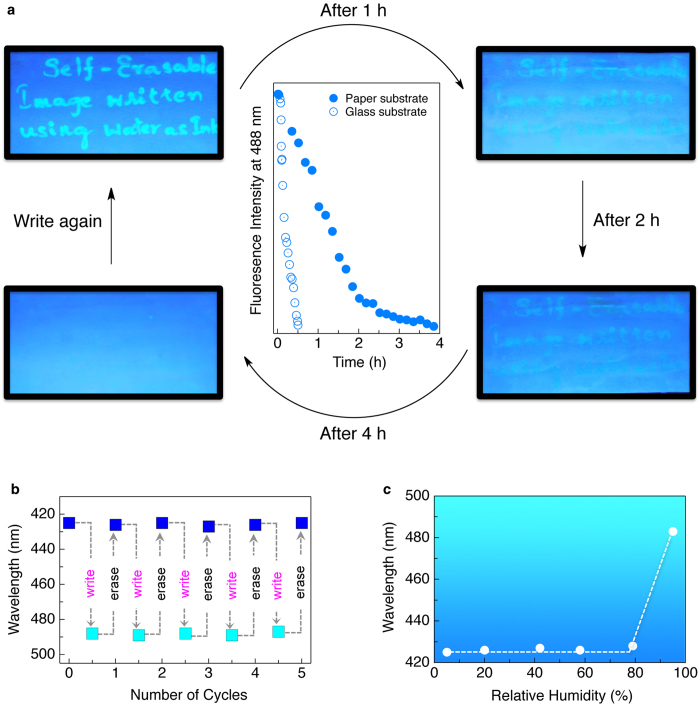
Use of water as a self-erasable fluorescent writing ink. (**a**) Photographs of hand written images on a **PE1** coated fluorescent paper under illumination at 365 nm over a period of 4 h. A secondary plot of the corresponding changes in the fluorescence spectra is shown in the middle. (**b**) Changes in the fluorescence colour of paper upon repeated cycles of writing with water and erasing with hot air. (**c**) Changes in the photoluminescence colour of paper upon exposure to different relative humidity.

In chloroform (*c* = 1 × 10^−3^ M) **PE1** showed a UV/Vis absorption maximum at 334 nm and an emission maximum at 395 nm (Supplementary Fig. S11). When this solution was coated on a paper and excited with a UV lamp (*λ*_ex_ = 365 nm), a blue emission with a maximum at 425 nm was observed. When brought in contact with water, the fluorescence colour of the paper changed from blue to intense cyan (*λ*_em_ = 488 nm) as shown in Fig. 1e. The cyan colour reverted to the original blue when the water was dried off. This process could be repeated a number of times (Fig. 2a and 2b) without significant photobleaching effect (Supplementary Fig. S12). The fluorescence colour change occurs only when the paper comes in contact with water or when the humidity of the surrounding reaches above 95% (Fig. 2c)^30^,^31^. The water-induced fluorescence switching property of **PE1** was observed on films prepared on different substrates also (Supplementary Fig. S13). Interestingly, the blue fluorescing chloroform solution of **PE2** (*c* = 1 × 10^−3^ M) when *c*oated on a paper immediately showed a cyan emission (Supplementary Fig. S14). On the other hand, a chloroform solution of **PE3** (*c* = 1 × 10^−3^ M) when coated on a paper showed blue fluorescence (Supplementary Fig. S14). Either the cyan emission of **PE2** or the blue emission of **PE3** did not show any change when brought in contact with water. These experiments revealed the fact that only **PE1** is capable of exhibiting the fluorescence colour change with water for which the presence of the hydrophilic oxyethylene chains and the amide hydrogen-bonding moiety are essential.

Having known the above water induced reversible fluorescence colour change of **PE1**, our next attempt was to explore the potential application of this molecule for self-erasable writing. For this purpose, ordinary writing papers (7.0 cm × 5.0 cm) were coated with a solution of **PE1** in chloroform (*c* = 1 × 10^−3^ M) and dried under vacuum for 30 min. The blue emission of the molecule remained intact on the paper, which was confirmed by illuminating with a UV lamp (*λ*_ex_ = 365 nm), however under daylight it looked like normal white paper. Upon writing on this paper using a pen filled with ordinary water under a UV light (365 nm) illumination, cyan letters were visible. The written paper after different time intervals is shown in the Fig. 2 a. The writing was clear to read up to 1h on keeping under ambient condition in an atmosphere having 80–85% humidity. The complete erasing of the writing occurred within 4 h, which is clear from the plot of the intensity of emission at 488 nm with time (Fig. 2a, solid circles). However, when the same experiment was performed on a glass plate coated with **PE1**, fast erasing was observed and complete disappearance of the letters happened within 20–25 min (Fig. 2a, open circles). The slow disappearance of the letters on paper substrates could be due to the better adherence of water molecules when compared to glass substrate. However, exposure to a hot air gun immediately erased the written letters on the paper substrates. These findings can be easily utilised to demonstrate free hand reversible writing on the **PE1** coated fluorescent paper using water as ink (Supplementary Fig. S15, Supplementary Movie S1). Detailed studies have revealed that the purity of water (presence of metal ions as well as different pH) has no substantial effect on the fluorescence response of the system. (Supplementary Fig. S16 and Fig. S17).

### Application as security labels

**Figure 3:**
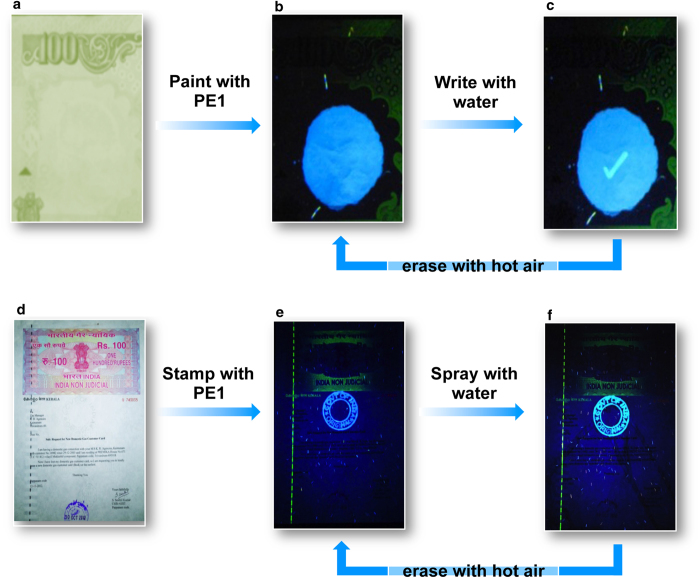
Water as a security marker for currency and documents. Photographs of (**a**) a part of a currency bill and (**d**) a document under normal light. Photographs taken under illumination at 365 nm (**b**) **PE1** coated currency bill, (**c**) tick mark made using water on **PE1** coated currency bill, (**e**) letters stamped over the document using **PE1** and (**f**) colour change after spraying water over it.

A practical application of the water responsive fluorescent molecular assembly of **PE1** is as a security label for checking the authenticity of currency and documents. Preventing currency counterfeit and document duplication are equally important as war against terrorism. Therefore, point of care authentication of currency and valuable documents has of great importance^12^,^13^,^14^,^32^,^33^,^34^. The currently used fluorescent labels on currency bills are reproducible and non-responsive to moisture or other stimuli. Therefore, water induced fluorescence colour change from blue to cyan with **PE1** assembly is a unique property that can be exploited for making security labels. The fluorescent assembly can be positioned on an appropriate place in the currency, which can be read as a blue emission. To check the authenticity, the blue fluorescent area needs to be touched with a wet finger or a mark should be made with water filled pen. At the point of contact with water, a bright cyan image appears which can be instantly erased with a hot air gun. This blue to cyan colour change and its instant reversal to the original blue fluorescence is the signature of authenticity (Fig. 3a-c). We further demonstrated the use of our fluorescent molecular assembly for securing a hundred rupees Indian non-judicial document paper (Fig. 3d-f). We made a stamp impression with letter written as “GOVT OF INDIA - ORIGINAL” over the document paper. The letters in the stamp impression shows a blue fluorescence under a UV lamp, which upon contact with moisture changed to cyan. The original blue fluorescence is regained after exposing to hot air. This simple, easy to use security system is difficult to duplicate since it is based on a subtle change in the molecular assembly on interaction with water, resulting the fluorescence modulation. This is evident from the fact that the closely resembling molecules **PE2** and **PE3** could not mimic the property of **PE1**. No other fluorescent molecular assemblies that respond to water through a blue to cyan fluorescence colour change are currently known.

**Figure 4:**
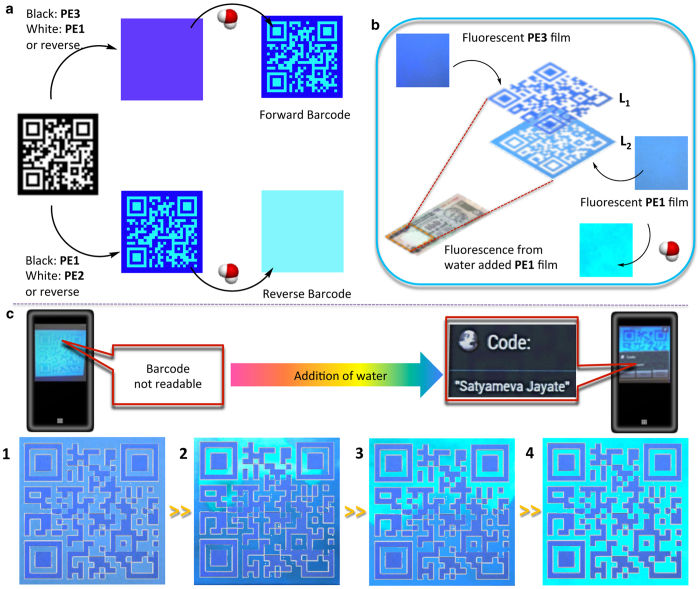
Water responsive hidden barcode as a super security feature. (**a**) Design principle of forward and reverse barcode using PE derivatives. (**b**) An ideal two-layer design of hidden barcode over a currency. Layers (L_1_ and L_2_) are composed of **PE3** and **PE1** respectively. (**c**) Simulated experiment to generate a forward barcode using **PE1** coated and **PE3** coated papers. The virtual barcode in the initial stages (1–3) remain undetected using a smart phone having barcode reader application. Upon complete wetting of **PE1** layer (4) a smart phone with a barcode reader (NeoReader) application can read the encoded message ‘Satyameva Jayate’ meaning ‘Truth Alone Triumphs’.

In order to further strengthen the security feature of our system, we envisaged barcodes with a three-stage identification protocol. The design of such hidden barcodes depends on control of optical contrast between the fluorescence colours being emitted from the black (binary digit 1) and white (binary digit 0) regions of a designed barcode. If the colour output from both the regions is nearly the same, the barcode remains undetected. Under an appropriate condition, if the fluorescence colour of one of the regions can be changed, the barcode becomes readable and embedded information could be revealed. A combination of the luminous changing **PE1** and the permanent blue emitting **PE3** can be used to generate a hidden forward barcode. In presence of water, **PE1** shows a cyan fluorescence and the barcode becomes readable. Similarly, **PE2**, which forms a cyan colour film, can be combined with **PE1** to generate a readable reverse barcode, which gets masked in presence of moisture (Fig. 4a). In order to establish the idea of barcoding, we carried out a simulated barcode experiment (Supplementary Fig. S18 and Fig. S19). For this purpose two independent films of **PE1** and **PE3** were prepared on filter papers. Water was dropcast on one of the edges to allow the filter paper to get wet. The changes in emission were recorded by using a camera. Multiple snapshots obtained from the individual films were used to prepare masks corresponding to the black region using **PE1** film and white region using **PE3** film at different time intervals. A combination of masks prepared from **PE1** and **PE3** films gave rise to a ‘virtual’ barcode in each case. Initially, this barcode was not readable since the pattern cannot be recognized by the barcode reader application installed in a smartphone (Fig. 4c1). Upon contact with water, the blue barcode pattern becomes visible in cyan colour background under a UV lamp (Fig. 4c2 and 4c3). This is the first manual step of the authenticity check. On complete wetting of the **PE1** layer, the barcode reader could read the pattern and decode the embedded information, which is the second step, which is an electronic reading (Fig. 4c4, Supplementary Movie S2). The final protocol is the drying of the barcode, which will temporarily mask the barcode information. In the case of a banknote the hylemetric information derived from the distribution of the fluorescent threads can be encoded inside the barcode, therefore serving as a hallmark for the central organisation that regulates the issue of banknotes as well as reducing its burden of excessive information storage. Each banknote or document with an individual ‘hidden barcode’ design makes the code unbreakable^33^. From our experiments, we could also confirm that these processes can be repeated any number of times. The barcode pattern recognition can be easily performed with any smart phone fitted with a UV LED and having the required mobile application (NeoReader) and hence can be performed at the point of care. It was also understood via proper simulation that such barcodes can also be prepared with any commercially available cyan or blue fluorescent ink so that the amount of stimuli responsive fluorescent ink (**PE1)** can be drastically brought down by a clever barcode design (Supplementary Fig. S20). A randomly located barcode defect site can be an added layer of protection. The overall concept of development of optical contrast has been demonstrated in the case of a barcode printed with normal ink having a defect site embedded with **PE1** and **PE3** films (Supplementary Fig. S21). Apart from this, we have demonstrated that the idea can be further extended to design optically masked logo of valuable products. Such ‘logos’ can be used for the one time verification of the authenticity of valuable objects, which can be tampered after use (Supplementary Movie S3).

### Mechanism of fluorescence colour change

**Figure 5:**
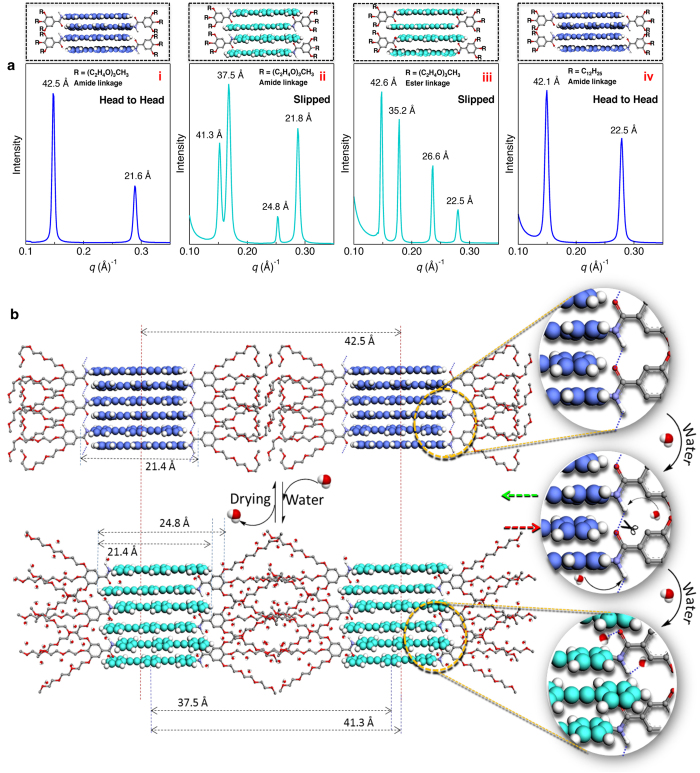
Mechanism of the fluorescence change based on molecular packing. (**a**) SAXS pattern of **PE1** in the (i) absence and (ii) presence of water, (iii) **PE2** and (iv) **PE3**. The corresponding molecular arrangements are shown on the top of the SAXS patterns. (**b**) Schematic illustration of sliding of the **PE1** molecule in the absence and presence of water on paper surface. Disruption of H-bonds and the breathing of the oxyethylene chains in presence of water experience an inward pushing of the molecules resulting in the change of an H-type (B-phase) to J-type (C-phase) packing. The images in panel ‘b’ (right) show the zoomed portion of the molecular arrangement illustrating the H-bond breaking and molecular sliding (arrows show the direction of sliding).

For an insight on the mechanism of the water induced fluorescence colour change, we performed film state small angle X-ray scattering (SAXS) analysis of the **PE1** molecular assembly before and after exposing to water. These data are compared with X-ray diffraction pattern of **PE2** and **PE3**. The initial blue emitting film of **PE1** (Fig. 5a, i) showed two sharp diffraction patterns at 42.5 and 21.6 Å, which are assigned to an H-type molecular arrangement. The 42.5 Å peak corresponds to the width of a single one-dimensional (1-D) layer of the molecules and the 21.6 Å peak corresponds to the rigid rod **PE** moiety. In THF, the emission arises from excitation of the monomer band at the 310–350 nm region. In the case of the film state, a strong blue shift in the excitation with red shift in emission was observed (Supplementary Fig. S22) indicating the formation of fluorescent H-type aggregates. After spraying water, the cyan emitting film showed four sharp diffractions 41.3, 37.5, 24.8 and 21.8 Å of varying intensities (Fig. 5a, ii). These peaks indicate the sliding of the molecules in the presence of water^35^,^36^ as depicted in (Fig. 5b, Supplementary Movie S4). The diffraction peak corresponding 24.8 Å may be associated with the slipped packing of the rigid **PE** moiety and 41.3 Å can be assigned to the total width of the 1-D assembly. The 21.8 and 37.5 Å peaks are assigned to the rigid **PE** part and the total length of the **PE1** molecule respectively. After complete removal of water, the regenerated blue emitting film exhibited the original X-ray pattern revealing the sliding back of the assembly to its original form. The X-ray diffraction pattern of **PE2** film (Fig. 5a, iii) showed four different peaks (42.6, 35.2, 26.6 and 22.5 Å) almost similar to that of the **PE1** film after water treatment, indicating identical slipped molecular packing. This slipped packing is formed due to the absence of hydrogen bonding amide groups in **PE2**. On the other hand, the **PE3** molecule (Fig. 5a, iv) having the alkyl chain exhibited diffraction patterns (42.1 and 22.5 Å) identical to that of the blue **PE1** film indicating the formation of hydrogen bonded H-type 1-D assembly in the film state.

A comparison of the diffraction patterns of **PE1**, **PE2** and **PE3** helped us in arriving at a plausible mechanistic pathway for the observed fluorescence variation of **PE1** when it comes in contact with water molecules as depicted in Fig. 5b. Our experimental data suggest a molecular slipping mechanism for the reversible fluorescence modulation. Interaction with water molecules facilitates breaking of the hydrogen bonds and the stretching of the hydrophilic ethoxy chains, which pushes the nearby molecules to the opposite directions along the inward direction. The presence of an amide bond as in **PE1** is essential for the observed reversible fluorescence colour change. In the blue phase (B-phase) each molecule is expected to form hydrogen bonds with the adjacent molecules through the amide groups as evident from the FT-IR spectral data (Supplementary Fig. S23). The absence of hydrogen bonding groups makes the molecules to pack in the slipped manner as observed in the case of **PE2** resulting in the cyan (C-phase). FT-IR spectra showed C=O stretching frequency of the B-phase and the C-phase respectively at 1670 cm^−1^ and 1661 cm^−1^ (Supplementary Fig. S23). When compared to the B-phase, C=O stretching frequency of the C-phase is shifted to a lower frequency, suggesting that the initial hydrogen bonds with amide groups weaken and the carbonyl groups enter into hydrogen bond with water molecules^37^, allowing the **PE1** molecules to pack in a slipped manner. Peaks corresponding to amide N-H stretching are not observed, because the peak corresponding to the O-H stretching of the water molecules are intense enough to hide the N-H stretching peaks.

## Discussion

Our fluorescent molecular assembly has several unique features required for an ideal security system. The “holy grail” of **PE1** is the initial blue fluorescence colour of the self-assembly since most of the blue emitting molecules either significantly quenches the fluorescence or shift the colour to longer wavelength upon self-assembly. Our molecular system not only possess good fluorescence quantum yield but also maintain its initial fluorescence colour in the film state. The fluorescence colour variation from blue to cyan occurs only with water on contact, and not with moist air, pressure or temperature. This molecular system has good photo and thermal stability, well suited for long-term application. Other structural variants of **PE1** do not exhibit blue to cyan fluorescence colour change when in contact with water and hence difficult to duplicate. Synthetic reproduction of the molecular system reported here involves several chemical steps, which can be repeated only with trained chemists in standard laboratory conditions. A molecular assembly having these features which can be used as a security label in combination with water induced slipping phenomenon is a unique example and not easily available. This system is needed only in small volumes for large area applications and adaptable to the protection of any paper based documents such as currencies, certificates, judicial stamp papers, and travel documents. This system can also be used either as colour changing tags or as barcode tags for one-time authenticity verification of valuable branded goods. These were possible by the logical combination of the power of molecular assemblies, sensitivity of fluorescence, the magical properties of water and the way to control them.

## Methods

### Preparation of fluorescent papers and security labels

Molecules **PE1-3**, were synthesized according to Supplementary Scheme S1 and Scheme S2 based on standard protocols. A solution was prepared by dissolving **PE1** (2.7 mg) in chloroform (3 ml) at room temperature. 2 ml of this solution (*c* = 1 × 10^−3^ M) was coated on paper strips (7 cm × 5 cm) and dried over a period of 30 min under vacuum. These paper strips were used as self-healing writing pads using a pen filled with ordinary water. Security labels were created over documents such as banknotes or stamped papers by coating the **PE1** solutions followed by drying under vacuum for 30 min. Over this layer a tick mark was made with a pen filled with ordinary water. The marked area showed a cyan colour whereas the untouched area appeared in blue upon illumination with a UV lamp (365 nm). After reading, the mark was erased on keeping for 3-4 h at room temperature or drying with hot air (maintained at ~70–80 °C) for 2 min. For printing over document papers, fluorescent ink was prepared by mixing 1 ml chloroform solution of **PE1** (*c* = 1 × 10^−3^ M) with 1.5 ml of polydimethylsiloxane (PDMS). This ink was used for creating impressions on documents using prefabricated stamps, which served as the security mark. Complete descriptions about various experimental techniques are provided in the supplementary information.

## Additional Information

**How to cite this article**: Thirumalai, R. *et al.* A slippery molecular assembly allows water as a self-erasable security marker. *Sci. Rep.* 5, 09842; doi: 10.1038/srep09842 (2015).

## Supplementary Material

Supplementary Movie S1

Supplementary Movie S2

Supplementary Movie S3

Supplementary Movie S4

Supplementary Information
