# Supplementary material for: A slippery molecular assembly allows water as a self-erasable security marker
Source: Sci Rep. 2015 May 5;5:9842. doi: 10.1038/srep09842 (PMC4419532; doi:10.1038/srep09842)
Supplement: Supplementary Information [file srep09842-s5.pdf]

A slippery molecular assembly allows water as a self-erasable security marker

Rajasekaran Thirumalai[1], Rahul Dev Mukhopadhyay[1,2], Vakayil K. Praveen[1] & Ayyappanpillai Ajayaghosh[1,2,*]

[1]Photosciences and Photonics Group, Chemical Sciences and Technology Division, CSIR-National Institute for Interdisciplinary Science and Technology (CSIR-NIIST), Trivandrum 695 019, India.

[2]Academy of Scientific and Innovative Research (AcSIR), CSIR-National Institute for Interdisciplinary Science and Technology (CSIR-NIIST), Trivandrum 695 019, India. *Correspondence and requests for materials should be addressed to A.A. (email: ajayaghosh@niist.res.in).

Index

1. Supplementary Methods . . . 3

    1.1. Synthesis . . . 3

        Scheme S1 . . . 4

        Scheme S2 . . . 9

    1.2. Measurements . . . 12

2. Supplementary Figures . . . 13

        Figure S1 . . . 13

        Figure S2 . . . 13

        Figure S3 . . . 14

        Figure S4 . . . 14

        Figure S5 . . . 15

        Figure S6 . . . 15

        Figure S7 . . . 16

        Figure S8 . . . 16

        Figure S9 . . . 17

        Figure S10 . . . 17

        Figure S11 . . . 18

        Figure S12 . . . 18

        Figure S13 . . . 19

        Figure S14 . . . 19

        Figure S15 . . . 20

        Figure S16 . . . 20

        Figure S17 . . . 21

        Figure S18 . . . 22

        Figure S19 . . . 23

        Figure S20 . . . 24

        Figure S21 . . . 25

        Figure S22 . . . 26

        Figure S23 . . . 27

Figure S24 29

3. Supplementary Table S1 28

4. Fluorescence Quantum Yield Measurements. 29

5. Supporting References 30

1. Supplementary Methods

1.1. Synthesis

Synthesis-general procedures. Unless otherwise stated, all starting materials and reagents were purchased from commercial suppliers and used without further purification. The solvents were purified and dried by standard methods prior to use. The reactions were monitored using thin layer chromatography (TLC) on silica gel 60 F_{254} (0.2 mm; Merck). Visualization was accomplished using UV lamp (365 nm). Column chromatography was performed on glass columns of different sizes hand packed with silica gel 60 (particle size 0.040–0.063 mm, Merck). Molecules **PE1-3**, were synthesized according to Supplementary Scheme S1 and Scheme S2 based on standard protocols.

Synthesis-characterization techniques. Melting points were determined with MEL-Temp-II melting point apparatus and are uncorrected. ^{1}H (300 and 500 MHz) and ^{13}C NMR (125 MHz) spectra were measured on a Bruker Avance DPX spectrometer. Chemical shifts are reported in parts per million (ppm) using tetramethylsilane (TMS) (δ_H = 0 ppm) or the solvent residual signal ($CDCl_3$: δ_C = 77.00 ppm) as an internal reference. The resonance multiplicity is described as s (singlet), d (doublet), t (triplet) and m (multiplet). IR spectra were recorded on a Perkin-Elmer Spectrum One FT-IR spectrometer using KBr sealed cell and plate for solution and film samples respectively. Mass spectra (MS) were recorded on a Thermo Scientific Q Exactive Hybrid

Quadrupole-Orbitrap electrospray ionization mass spectrometer (ESI-MS) or a JEOL JSM 600 fast atom bombardment (FAB) high-resolution mass spectrometer.

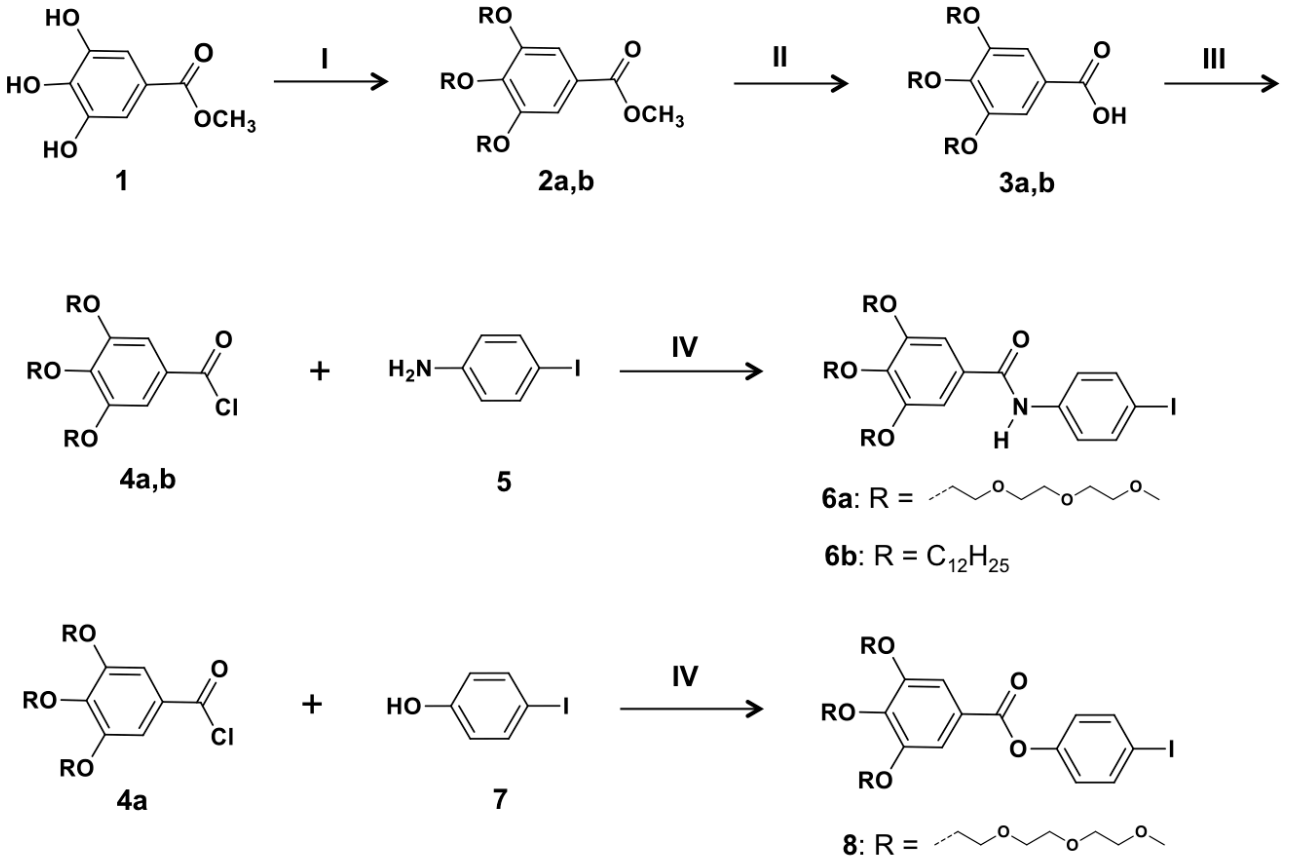

Scheme S1 | Synthesis of compounds 2-8. Reagents and conditions: I) Triethylene glycol monomethyl ether tosylate, K_2CO_3, CH_3CN, 80 ^oC, 48 h for **2a** and $C_{12}H_{25}Br$, K_2CO_3, DMF, 100 ^oC, 24 h for **2b**; II) KOH, EtOH, 80 ^oC, 12 h; III) $SOCl_2$, CH_2Cl_2, rt., 5 h; IV) toluene, Et_3N, rt., 12 h.

Synthesis of methyl 3,4,5-tris(2-(2-(2-methoxyethoxy)ethoxy)ethoxy)benzoate (2a): Compound **1** (1.0 g, 5.4 mmol) and K_2CO_3 (3.8 g, 27.0 mmol) were taken in a 250 mL two-neck round bottom flask containing 40 mL dry acetonitrile. The mixture was stirred at room temperature for 30 minutes and triethylene glycol monomethyl ether tosylate (6.0 g, 19.0 mmol) was added dropwise. The reaction mixture was stirred at 80 °C for 48 h. After cooling the reaction mixture to room temperature, the solvent was evaporated under reduced pressure. The residue thus obtained

extracted using chloroform, washed with water, brine and dried over anhydrous sodium sulphate. The crude product was subjected to column chromatography (2% methanol/chloroform) over silica gel that gave the pure product.

Yield: 90%; ^{1}H NMR (500 MHz, $CDCl_3$): δ = 7.26 (s, 2H), 4.20-4.15 (m, 6H), 3.85 (s, 3H), 3.83-3.77 (m, 6H), 3.76-3.70 (m, 6H), 3.69-3.62 (m, 12H), 3.61-3.58 (m, 6H), 3.50 (s, 3H), 3.34 (s, 6H) ppm; HRMS-FAB (m/z): $[M+Na]^+$ calcd. for $C_{29}H_{50}O_{14}$, 645.32; found, 645.70.

Synthesis of methyl 3,4,5-tris(dodecyloxy)benzoate (2b): Compound **1** (3.0 g, 16.3 mmol) and K_2CO_3 (13.5 g, 98 mmol) were taken in a 250 mL two-neck round bottom flask containing 60 mL dry DMF. The mixture was stirred at room temperature for 30 minutes and then 1-bromododecane (13.0 g, 52.0 mmol) was added dropwise. The reaction mixture was stirred at 80 °C for 24 h. After cooling to room temperature, the reaction mixture was poured into water and extracted three times with chloroform. The combined organic layer was washed with brine and water, dried over anhydrous sodium sulphate. After removal of the solvent under reduced pressure, the residue was subjected to column chromatography (5% ethylacetate/hexane) over silica gel afforded the pure product.

Yield: 80%; ^{1}H NMR (500 MHz, $CDCl_3$): δ = 7.25 (s, 2H), 4.03-3.99 (m, 6H), 3.89 (s, 3H), 1.84-1.81 (m, 6H), 1.50-1.45 (m, 6H), 1.35-1.27 (m, 48H), 0.89-0.88 (t, 9H) ppm; HRMS-FAB (m/z): $[M+1]^+$ calcd. for $C_{44}H_{80}O_5$, 689.60; found, 690.33.

Synthesis of 3,4,5-tris(2-(2-(2-methoxyethoxy)ethoxy)ethoxy)benzoic acid (3a): Compound **2a** (3 g, 4.8 mmol) was taken in a 250 mL round bottom flask containing 50 mL ethanol and 10 ml 0.5 M KOH in ethanol was added to it. The reaction mixture was heated to 80 °C for 12 h and after cooling to room temperature the solvent was evaporated. The residue was extracted using chloroform and shake well with 10% HCl. The organic layer was washed with water, brine, dried over anhydrous sodium sulphate and then solvent was evaporated under reduced pressure to get the crude product. This was used for next step without further purification.

Yield: 90%; ^1H NMR (500 MHz, $CDCl_3$): δ = 7.33 (s, 2H), 4.26-4.19 (m, 6H), 3.89-3.81 (m, 6H), 3.76-3.72 (m, 6H), 3.69-3.63 (m, 12H), 3.57-3.54 (m, 6H), 3.38 (s, 9H) ppm; HRMS-FAB (m/z): $[M+Na]^+$ calcd. for $C_{28}H_{48}O_{14}$, 631.67; found, 631.62.

Synthesis of 3,4,5-tris(dodecyloxy)benzoic acid (3b): Compound **2b** (5 g, 7.27 mmol) was taken in a 250 mL round bottom flask containing 60 mL ethanol. 20 ml 0.5 M KOH in ethanol was added to it. The reaction mixture was heated to 80 °C for 12 h and after cooling to room temperature, it was poured into a beaker containing cold dilute HCl. The white colour precipitate formed was filtered and dried under vacuum. The product was used for next step without further purification.

Yield: 90%; ^1H NMR (500 MHz, $CDCl_3$): δ = 7.34 (s, 2H), 4.08-4.03 (m, 6H), 1.87-1.74 (m, 6H), 1.52-1.47 (m, 6H), 1.35-1.27 (m, 48H), 0.91-0.88 (t, 9H) ppm; HRMS-FAB (m/z): $[M+1]^+$ calcd. for $C_{43}H_{78}O_5$, 674.58; found, 674.33.

Synthesis of 3,4,5-tris(2-(2-(2-methoxyethoxy)ethoxy)ethoxy)benzoyl chloride (4a): Compound **3a** (1 g, 1.64 mmol) was taken in a two-neck round bottom flask containing 20 ml dry dichloromethane under nitrogen atmosphere. $SOCl_2$ (0.5 g, 5.0 mmol) was added dropwise through a syringe. The reaction mixture was allowed to stir at room temperature for 5 h and then purged with nitrogen to remove the solvent and unreacted $SOCl_2$. The residue obtained was used for the next step without purification and characterization.

Synthesis of 3,4,5-tris(dodecyloxy)benzoyl chloride (4b): Compound **3b** (3 g, 4.4 mmol) was taken in a two neck round bottom flask containing 20 ml dry dichloromethane under nitrogen atmosphere. $SOCl_2$ (1.6 g, 13.3 mmol) was added dropwise through a syringe. The reaction mixture was allowed to stir at room temperature for 5 h and then purged with argon to remove the solvent and unreacted $SOCl_2$. The residue obtained was used for the next step without purification and characterization.

Synthesis of *N*-(4-iodophenyl)-3,4,5-tris(2-(2-(2-methoxyethoxy)ethoxy)ethoxy) benzamide (6a): Iodoaniline **5** (0.44 g, 2.0 mmol) was dissolved in 20 ml dry toluene in a two neck round bottom flask under nitrogen atmosphere. Dry triethylamine (2 ml) was added to the flask and allowed the reaction mixture to stir at room temperature for 15 min. Compound **4a** was dissolved in 10 ml dry toluene separately and added to the reaction flask dropwise. The reaction mixture was then allowed to stir at room temperature for 12 h. After completion of reaction, the solvent toluene was evaporated and the residue was extracted using chloroform. The organic layer was washed with water, brine and then dried over anhydrous sodium sulphate. After the removal of solvent, residue was purified by silica gel column chromatography using 5% methanol/chloroform as an eluent.

Yield: 75%; ^{1}H NMR (500 MHz, $CDCl_3$): δ = 8.74 (s, 1H), 7.64 (d, J = 8.5 Hz, 2H), 7.51 (d, J = 9.0 Hz, 2H), 7.22 (s, 2H), 4.21-4.19 (m, 6H), 3.83-3.79 (m, 6H), 3.76-3.72 (m, 6H), 3.69-3.63 (m, 12H), 3.53-3.50 (m, 6H), 3.37 (s, 3H), 3.32 (s, 6H) ppm; HRMS-FAB (m/z): $[M+Na]^+$ calcd. for $C_{34}H_{52}INO_{13}$,832.25; found, 832.75.

Synthesis of 3,4,5-tris(dodecyloxy)-N-(4-iodophenyl)benzamide (6b): Iodoaniline **5** (1.3 g, 5.2 mmol) was dissolved in 20 ml dry toluene in a two-neck round bottom flask under nitrogen atmosphere. Dry triethylamine (2 ml) was added to the flask and allowed the reaction mixture to stir at room temperature for 15 min. Compound **4b** was dissolved in 10 ml dry toluene separately and added to the reaction flask dropwise. The reaction mixture was allowed to stir at room temperature for 12 h. After completion of reaction, solvent toluene was evaporated and the residue was extracted using chloroform. The organic layer was washed with water, brine and then dried over anhydrous sodium sulphate. After the removal of solvent, residue was purified by silica gel column chromatography using 5% ethylacetate/hexane as an eluent.

Yield: 70%; ^1H NMR (300 MHz, $CDCl_3$): δ = 7.78 (s, 1H), 7.67 (d, J = 8.7 Hz, 2H), 7.44 (d, J = 8.7 Hz, 2H), 7.01 (s, 2H), 4.02-3.98 (m, 6H), 1.83-1.70 (m, 6H), 1.52-1.47 (m, 6H), 1.35-1.27 (m, 48H), 0.91-0.86 (t, 9H) ppm; HRMS-FAB (m/z): $[M+1]^+$ calcd. for $C_{49}H_{82}INO_4$, 876.53; found, 877.27.

Synthesis of 4-iodophenyl 3,4,5-tris(2-(2-(2-methoxyethoxy)ethoxy)ethoxy) benzoate (8): Iodophenol **7** (0.44 g, 2.0 mmol) was dissolved in 20 ml dry toluene in 250 ml two neck round bottom flask under nitrogen atmosphere. Dry triethylamine (2 ml) was added to the flask and allowed the reaction mixture to stir at room temperature for 15 min. Compound **4a** was dissolved in 10 ml dry toluene separately and added to the reaction flask dropwise. The reaction mixture was then allowed to stir at room temperature for 12 h. After completion of reaction, solvent toluene was evaporated under reduced pressure and the residue was extracted using chloroform. The organic layer was washed with water, brine and then dried over anhydrous sodium sulphate. After the removal of solvent, residue was purified by silica gel column chromatography using 5% methanol/chloroform as an eluent.

Yield: 75%; ^1H NMR (500 MHz, $CDCl_3$): δ = 7.74 (d, J = 8.5 Hz, 2H), 7.43 (s, 2H), 6.98 (d, J = 8.5 Hz, 2H), 4.28-4.22 (m, 6H), 3.89-3.82 (m, 6H), 3.76-3.72 (m, 6H), 3.69-3.63 (m, 12H), 3.56-3.53 (m, 6H), 3.38 (s, 3H), 3.37 (s, 6H) ppm; HRMS-FAB (m/z): $[M+Na]^+$ calcd. for $C_{34}H_{51}IO_{14}$,833.23; found, 833.22.

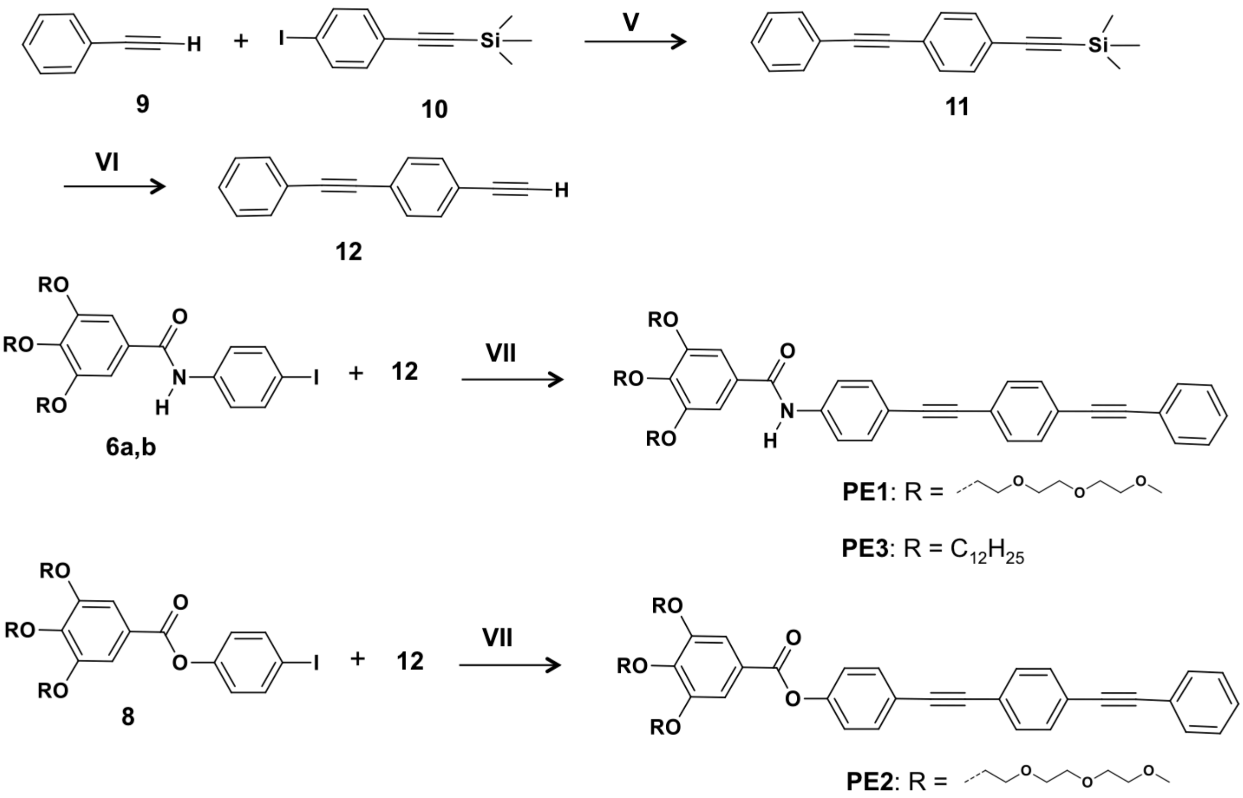

Scheme S2 | Synthesis of PE1, PE2 and PE3. Reagents and conditions: V) $PdCl_2(PPh_3)_2$, CuI, THF, Et_3N, rt., 12 h; VI) KF, CH_2Cl_2, CH_3OH, rt., 6 h; VII) $PdCl_2(PPh_3)_2$, CuI, THF, Et_3N, rt., 24 h.

.

Synthesis of trimethyl((4-(phenylethynyl)phenyl)ethynyl)silane (11): In a two neck round bottom flask purged with argon, ((4-iodophenyl)ethynyl)trimethylsilane **10** (0.5 g, 1.67 mmol), $Pd(PPh_3)_2Cl_2$ (0.1 g, 0.17 mmol) and CuI (0.03 g, 0.1 mmol) were dissolved in a degassed 20 ml (1:1) mixture of triethylamine and THF. Phenylacetylene **9** (0.2 g, 2.0 mmol) was added to the reaction mixture and stirred at room temperature under argon atmosphere for 12 h. After completion of reaction, chloroform (50 mL) was added to the reaction mixture and then stirred well with 10% HCl to neutralize triethylamine. The organic layer was washed with water, brine and then dried over anhydrous sodium sulphate. After the removal of solvent, residue was purified by silica gel column chromatography using 2% ethylacetate/hexane as an eluent.

Yield: 92%; ^{1}H NMR (500 MHz, $CDCl_3$): δ = 7.53-7.51 (m, 2H), 7.47-7.43 (m, 4H), 7.36-7.34 (m, 3H), 1.55 (s, 9H) ppm; HRMS-FAB (m/z): $[M]^+$ calcd. for $C_{19}H_{18}Si$, 274.12; found, 274.71.

Synthesis of 1-ethynyl-4-(phenylethynyl)benzene (12): To a solution of **11** (0.42 g, 1.71 mmol) in 5 ml dichloromethane, KF (1.0 g, 17.0 mmol) in 15 ml methanol was added to it and allowed to stir at room temperature for 6 h. After completion of the reaction, the reaction mixture was extracted using chloroform, washed with water, brine and then dried over anhydrous sodium sulphate. Solvent was evaporated under reduced pressure and the residue was used for the next step without further purification.

Yield: 93%; ^{1}H NMR (500 MHz, $CDCl_3$): δ =7.54-7.52 (m, 2H), 7.50-7.46 (m, 4H), 7.37-7.35 (m, 3H), 3.17 (s, 1H) ppm; HRMS-FAB (m/z): $[M]^+$ calcd. for C_6H_{10}, 202.08; found, 202.49.

Synthesis and characterization of PE derivatives. In a general synthetic procedure, the aryl halide (0.80 mmol), bis(triphenylphosphine)palladium (II) dichloride (10 mol%), and copper (I) iodide (10 mol%) were added to an oven-dried two neck round bottom flask equipped with a magnetic stirring bar. The round bottom flask was then sealed with a rubber septum, evacuated and backfilled with argon three times. Degassed triethylamine (10 ml) was added followed by degassed THF (10 ml) to serve as the co-solvent. After stirring for 5 minutes at room temperature, the terminal alkyne 1-ethynyl-4-(phenylethynyl)benzene (0.96 mmol) dissolved in 10 ml (1:1) mixture of degassed triethylamine and THF was added and the reaction mixture was stirred at room temperature until complete reaction was noted by the TLC. The reaction mixture was extracted using chloroform and washed with dilute hydrochloric acid. The organic layer was washed with brine and dried over anhydrous sodium sulphate and then evaporated under reduced pressure. The crude product was then purified by column chromatography using silica gel as adsorbent.

PE1: Yield, 57%; m.p.: 67-69 °C; TLC ($CHCl_3$:MeOH, 95:5 v/v): R_f = 0.48; ^{1}H NMR (500 MHz, $CDCl_3$): δ = 8.64 (s, 1H), 7.73 (d, J = 8.5 Hz, 2H), 7.55-7.7.51 (m, 8H), 7.36-7.35 (m, 3H), 7.25 (s, 2H), 4.25-4.23 (m, 6H), 3.85-3.80 (m, 6H), 3.73-3.71 (m, 6H), 3.68-3.63 (m, 12H), 3.53-3.51 (m, 6H), 3.38 (s, 3H), 3.32 (s, 6H) ppm; ^{13}C NMR (125 MHz, $CDCl_3$): δ = 165.5, 152.5, 142.2, 138.8, 132.3, 131.6, 131.5, 131.44, 129.9, 128.4, 128.3, 123.2, 123.1, 122.9, 120.1, 118.43, 107.9, 91.3, 91.2, 89.1, 88.8, 72.3, 71.9, 71.8, 70.7, 70.6, 70.6, 70.4, 69.82, 69.19, 58.9, 58.2 ppm; IR (KBr): 3240, 2874, 2208, 1667, 1585 cm^{-1}; ESI-MS (m/z): $[M+Na]^+$ calcd. For $C_{50}H_{61}NO_{13}$, 906.41; found, 906.40.

PE2: Yield, 60%; m.p.: 58-60 °C; TLC ($CHCl_3$:MeOH, 95:5 v/v): R_f = 0.52; ^{1}H NMR (500 MHz, $CDCl_3$): δ = 7.60 (d, J = 8.5 Hz, 2H), 7.55-7.52 (m, 6H), 7.45 (s, 2H), 7.37-7.35 (m, 3H), 7.21 (d, J = 8.5 Hz, 2H), 4.28-4.24 (m, 6H), 3.89-3.82 (m, 6H), 3.76-3.72 (m, 6H), 3.69-3.63 (m, 12H), 3.56-3.53 (m, 6H), 3.37 (s, 3H), 3.36 (s, 6H) ppm; ^{13}C NMR (125 MHz, $CDCl_3$): δ = 163.4, 151.5, 150.1, 142.4, 131.9, 130.6, 130.5, 127.5, 127.4, 122.9, 122.2, 122.1, 121.9, 121.0, 108.8, 98.9, 90.28, 89.5, 88.25, 88.1, 71.7, 71.2, 70.0, 69.8, 69.7, 68.8, 68.14, 58.18 ppm; IR (KBr): 2876, 2212, 1734, 1587 cm^{-1}; ESI-MS (m/z): $[M+Na]^+$ calcd. for $C_{50}H_{60}O_{14}$, 907.40; found, 907.39.

PE3: Yield, 48%; m.p.: 114-116 °C; TLC (*n*-hexane:$CHCl_3$, 1:1 v/v): R_f = 0.55; ^{1}H NMR (500 MHz, $CDCl_3$): δ = 7.77 (s, 1H), 7.66 (d, J = 9.0 Hz, 2H), 7.55-7.51 (m, 8H), 7.37-7.35 (m, 3H), 7.04 (s, 2H), 4.05-4.01 (m, 6H), 1.83-1.70 (m, 6H), 1.52-1.47 (m, 6H), 1.35-1.27 (m, 48H), 0.90-0.88 (t, 9H) ppm; ^{13}C NMR (125 MHz, $CDCl_3$): δ = 165.2, 154.5, 142.3, 137.5, 135.2, 132.4, 129.7, 128.5, 127.6, 126.7, 125.2, 118.7, 105.6, 90.2, 71.2, 69.5, 32.2, 28.9, 27.2, 25.3, 22.1, 15.7 ppm. IR (KBr): 3255, 2920, 2852, 2214, 1647, 1583 cm^{-1}; ESI-MS (m/z): $[M+1]^+$ calcd. for $C_{65}H_{91}NO_4$, 950.69; found, 950.

1.2. Measurements

Optical measurements. The solvents for the spectroscopic measurements are spectroscopic grade (99.8 %) and were used as received. The UV/Vis absorption spectra were recorded on a Shimadzu spectrophotometer UV-2100. The emission spectra were recorded on a SPEX-Flourolog-3 FL3-221 spectrofluorimeter using a front face sample holder. In matrix scan experiment, fluorescence spectra were recorded at different excitation wavelengths between 250 and 400 nm. The corresponding emission was collected between 350 and 670 nm. The result was shown as a contour plot based on emission intensity of the spectra at different excitation wavelengths. Fluorescence lifetimes were measured using IBH (FluoroCube) time-correlated picosecond single photon counting (TCSPC) system.

Morphological characterization. Scanning electron microscopy (SEM) images were obtained from a Zeiss EVO 18 cryo SEM Special Edn with variable pressure detector working at 20-30 kV. Transmission electron microscopy (TEM) was performed on a FEI, TECNAI 30 G2 S-TWIN microscope at an accelerating voltage of 100 kV. Fluorescence microscopy images were taken using a Leica DFC 290 Microscope. Dynamic light scattering (DLS) analyses were carried out with a Zetasizer Nano S from Malvern Instruments.

X-ray diffraction. Samples for the XRD studies were prepared by transferring chloroform solution of PE derivatives (5 mg/mL) onto glass plates (thickness: 0.05 mm), dried slowly to evaporate the solvent, and finally dried under vacuum. The X-ray diffraction pattern was recorded at ambient conditions using PANalytical 3 kW X'pert PRO diffractometer. The samples were irradiated using a monochromatic CuKa X-ray (wavelength, λ = 1.54 Å). In the case of **PE1** derivative, the blue emitting film after recording XRD was exposed to moisture to get the cyan emitting film and then recorded the diffraction pattern. The cyan-emitting film was then exposed to hot air and diffraction pattern of the re-generated blue-emitting film was recorded.

2. Supplementary Figures

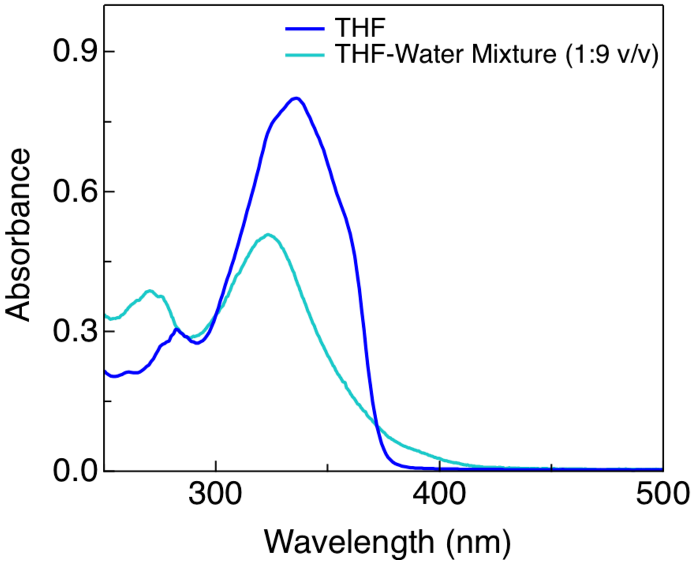

Supplementary Figure S1 | Optical characterization of PE1 aggregate. Absorption spectra of **PE1** (c = 1 × 10^{-5} M) in THF and THF-water mixture (1:9 v/v).

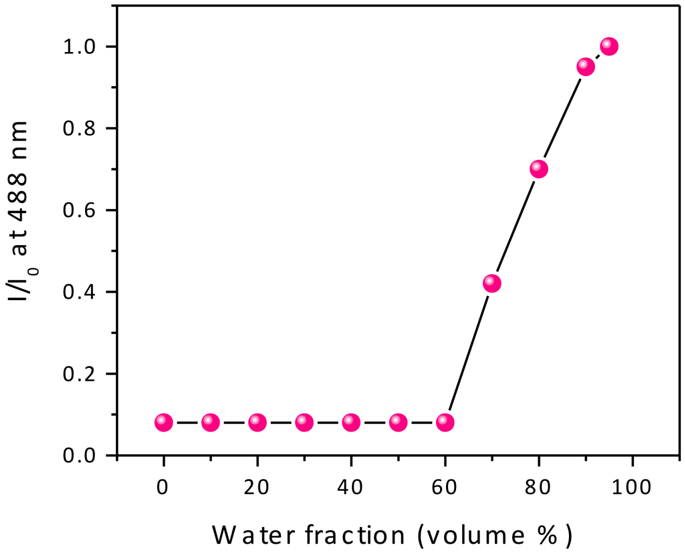

Supplementary Figure S2 | Optical characterization of PE1 aggregates. Changes in fluorescence intensities of **PE1** with different water fractions in the THF-water mixtures.

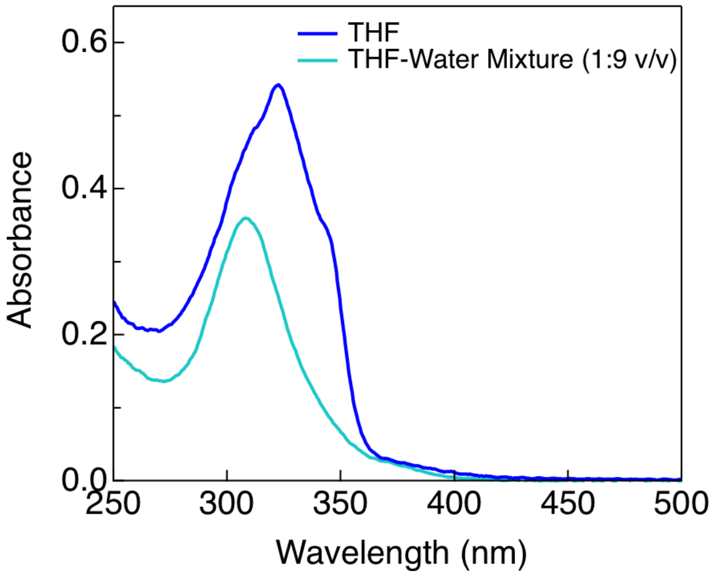

Supplementary Figure S3 | Optical characterization of PE2 aggregates. Absorption spectra of **PE2** (c = 1 × 10^{-5} M) in THF and THF-water mixture (1:9 v/v).

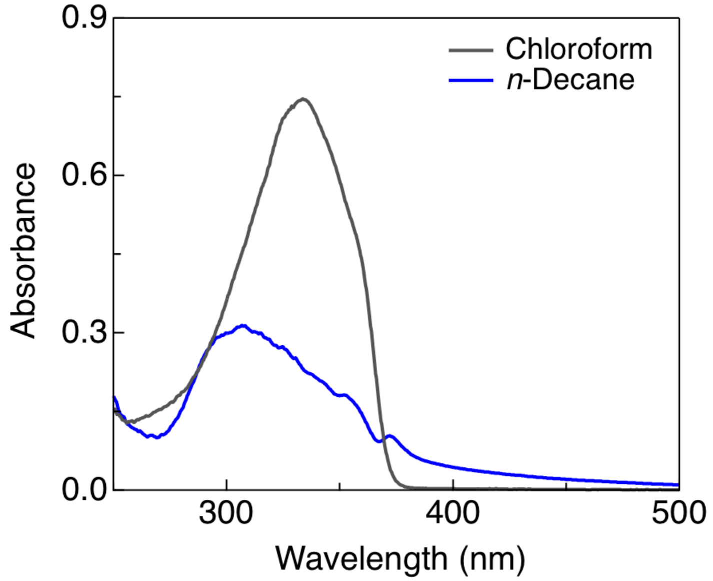

Supplementary Figure S4 | Optical characterization of PE3 aggregates. Absorption spectra of **PE3** (c = 1 × 10^{-4} M) in chloroform and *n*-decane.

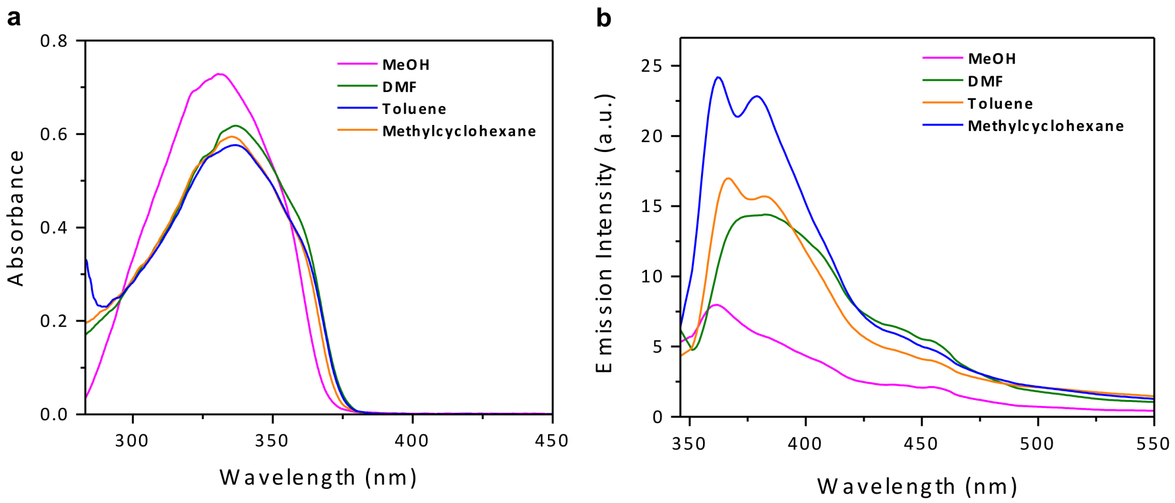

Supplementary Figure S5 | Photophysical characterization of PE1. (a) Absorption and (b) emission spectra (λ_{ex} = 340 nm) of **PE1** (1 × 10^{-5} M) in different solvents.

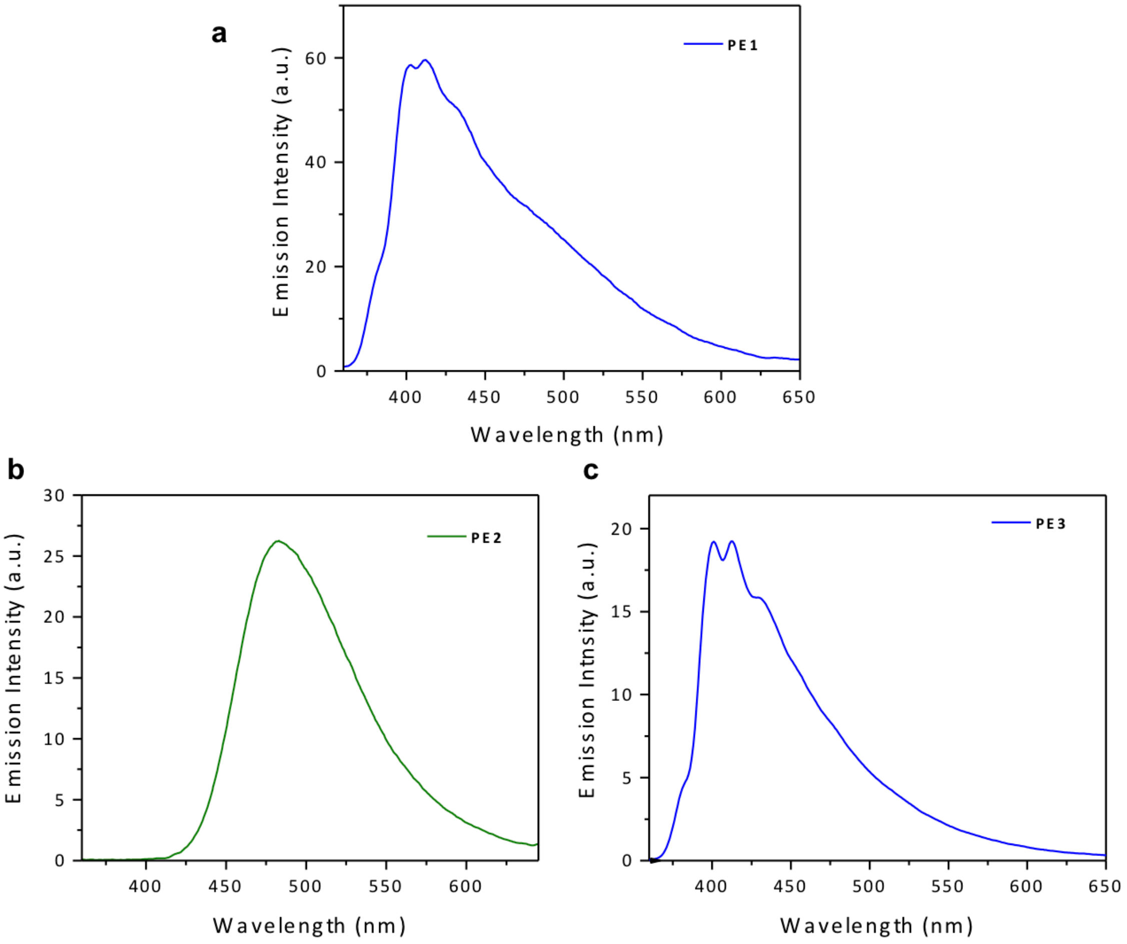

Supplementary Figure S6 | Fluorescence properties of PE derivatives. Emission spectra in the powder form (a) **PE1** (b) **PE2** and (c) **PE3** (λ_{ex} = 340 nm).

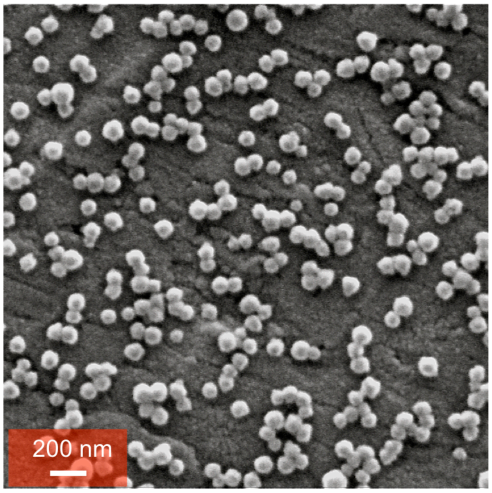

Supplementary Figure S7 | Morphology of PE1 aggregates. SEM image of **PE1** aggregates prepared from THF-water mixture (1:9 v/v), $c = 5 \times 10^{-5}$ M displaying spherical particles.

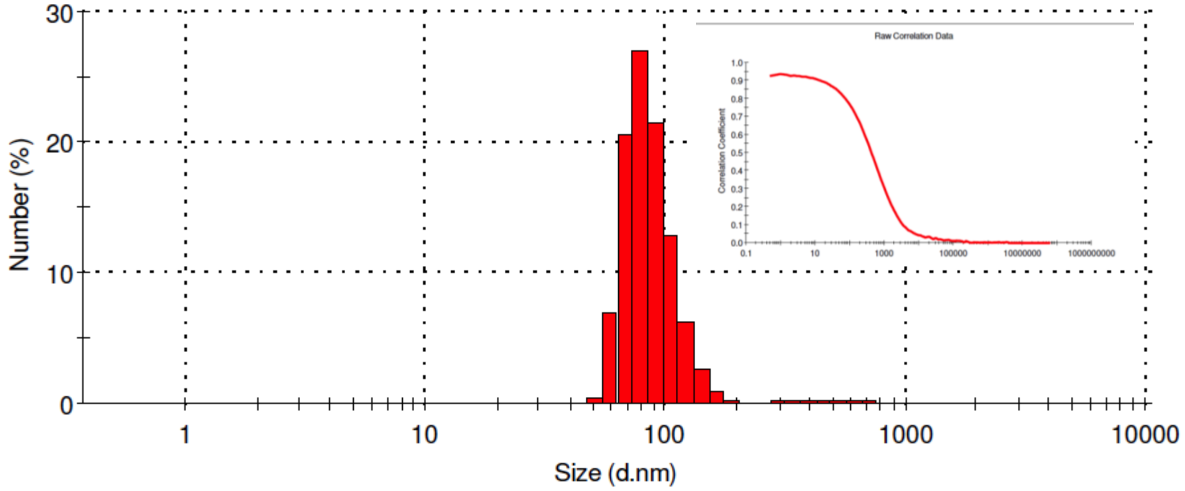

Supplementary Figure S8 | DLS profile of PE1 aggregates. DLS data shows the formation spherical particles in THF-water mixture (1:9 v/v) at 5 x 10^{-5} M concentration with average size 122 nm. Inset shows the correlogram data.

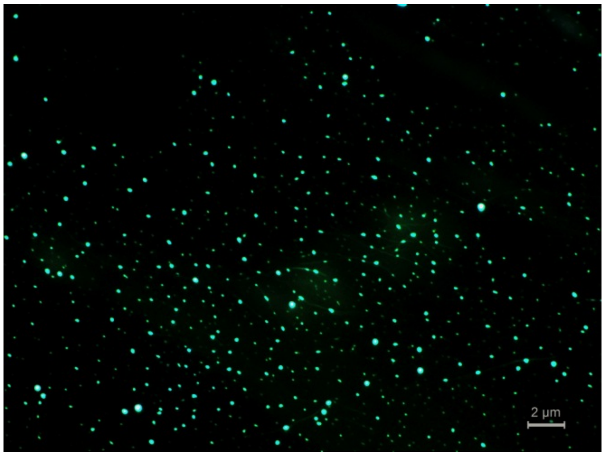

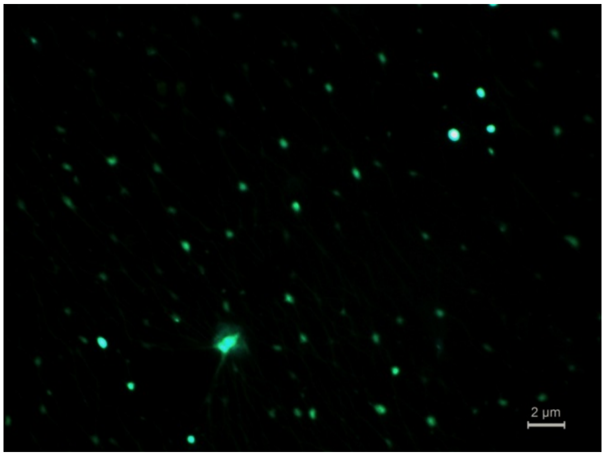

Supplementary Figure S9 | Fluorescence microscopy images of PE1. The images show the particles formed from **PE1** (c = 5 x 10^{-5} M) in THF-Water (1:9 v/v) are fluorescent. The samples prepared by drop casting a drop of solution over glass plate and allowed it to dry in ambient temperature. Scale bar is 2 μm.

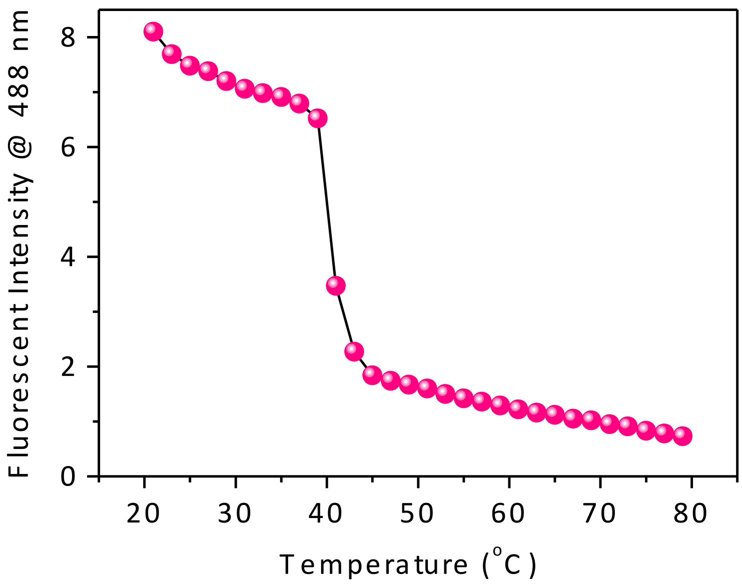

Supplementary Figure S10 | Stability of spherical particles. The plot of fluorescence intensity monitored at 488 nm versus temperature shows the stability of spherical particles formed from **PE1** (c = 5.0 × 10^{-5} M) in THF-water mixture (1:9 v/v).

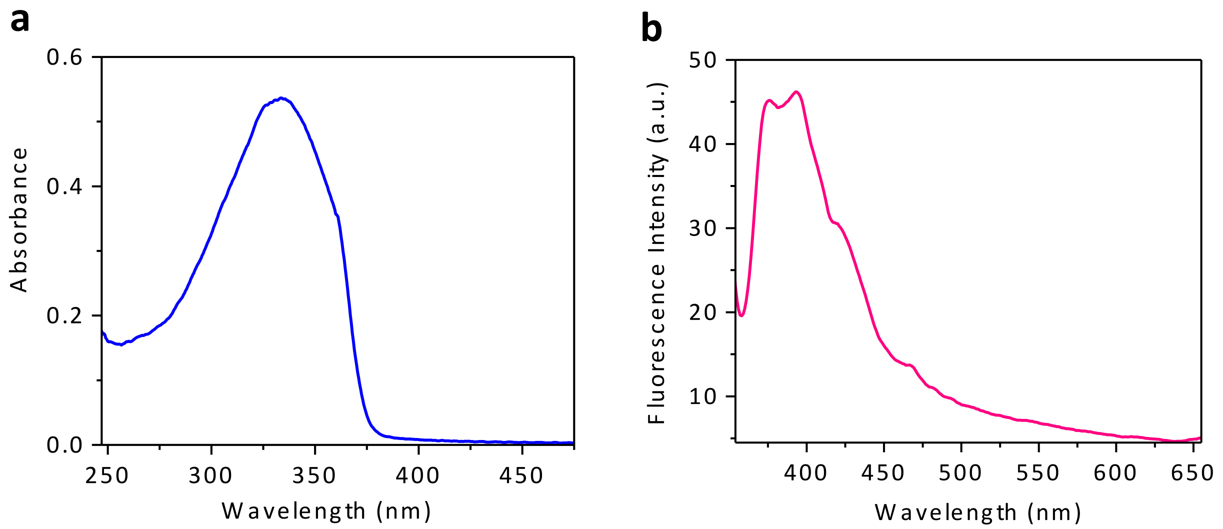

Supplementary Figure S11 | Optical features of PE1 in chloroform. (a) Absorption and (b) emission spectra (λ_{ex} = 340 nm) of **PE1** (1×10^{-3} M) in chloroform.

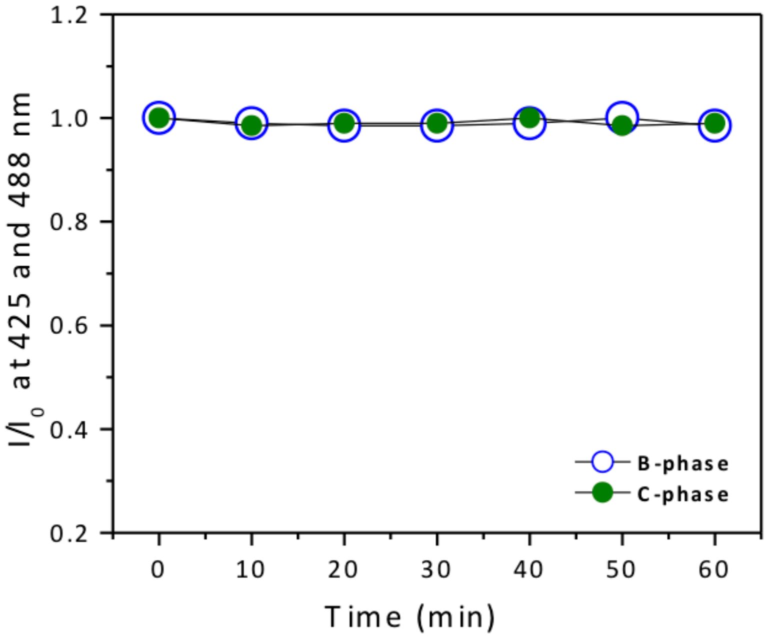

Supplementary Figure S12 | Photostability of PE1 in B-Phase and C-Phase. Comparison of variation of emission in the B-Phase and C-Phase of **PE1** coated over the quartz plate under ambient light. Emission of B-phase and C-phase monitored at 425 and 488 nm respectively. To main the C-phase minimal quantity of water was sprayed from time to time. The same experiment can be repeated over several days using the same substrate.

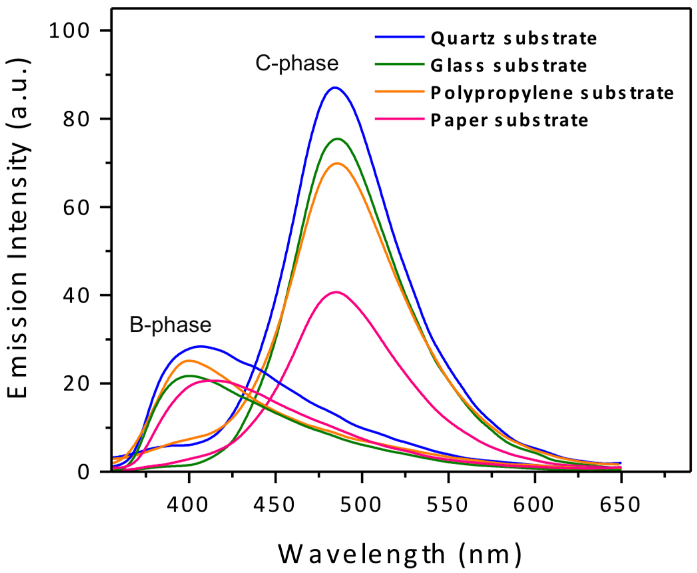

Supplementary Figure S13 | Effect of solid support on the fluorescence of PE1. Emission spectra (λ_{ex} = 340 nm) of **PE1** in B-phase and C-phase drop casted over different solid substrates.

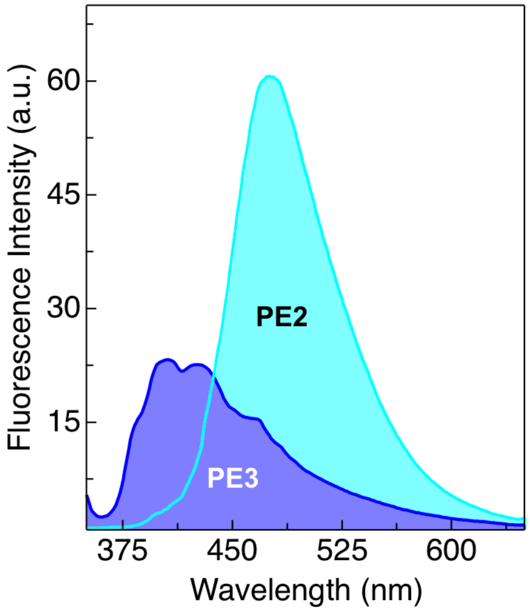

Supplementary Figure S14 | Film state emission properties of PE2 and PE3. Emission spectra (λ_{ex} = 340 nm) of **PE2** (cyan) and **PE3** (blue) coated paper. **PE2** exhibited the inherent blue emission on paper whereas **PE3** coated paper showed a shift of the blue emission to cyan even in the absence of water. Either the cyan emission of **PE2** or the blue emission of **PE3** did not show any change when brought in contact with water. For coating on paper, PE solutions were prepared in chloroform ($c = 1 \times 10^{-3}$ M).

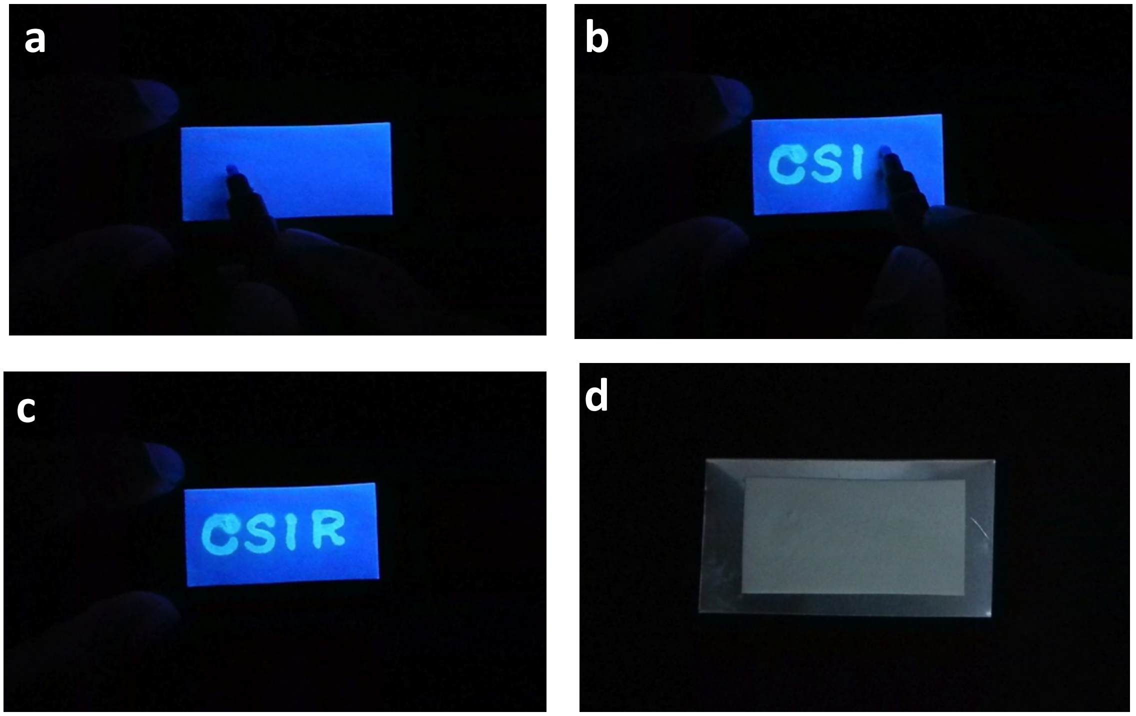

Supplementary Figure S15 | Snap shots of the writing images. The figures show self-erasable writing on **PE1** coated fluorescent paper under UV light (365 nm). (a) Film before writing (b) and (c) writing of images (see movie for the actual process). (d) The written image under normal daylight (note that the written images are not visible).

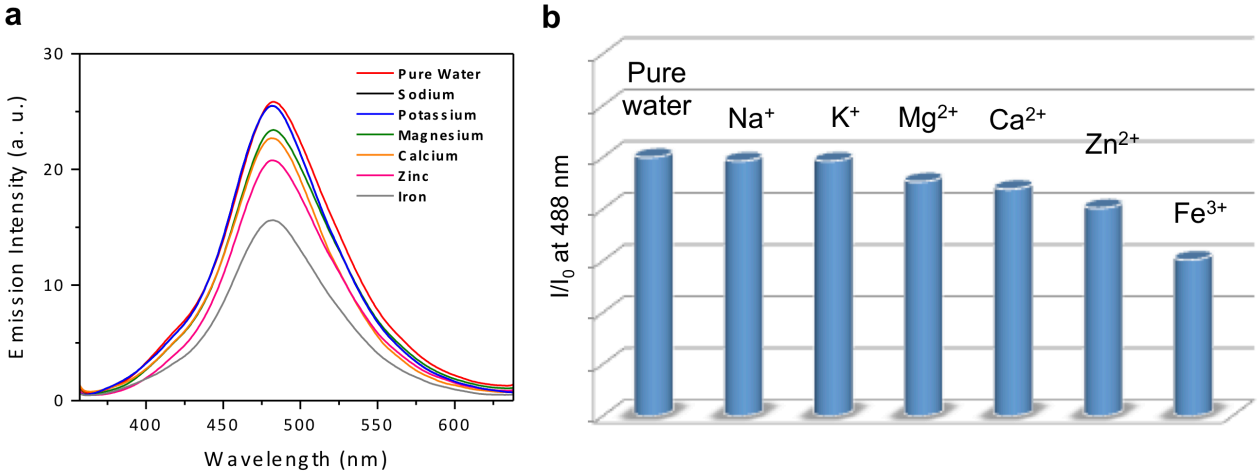

Supplementary Figure S16 | Effect of metal ion. (a) Fluorescence changes in **PE1** coated over paper substrate on addition of water having different metal ions (c = 1 x 10^{-3} M). (b) Bar diagram representation of fluorescence intensity changes monitored at 488 nm. Fluorescence intensity obtained with application of deionised water is taken as I_0.

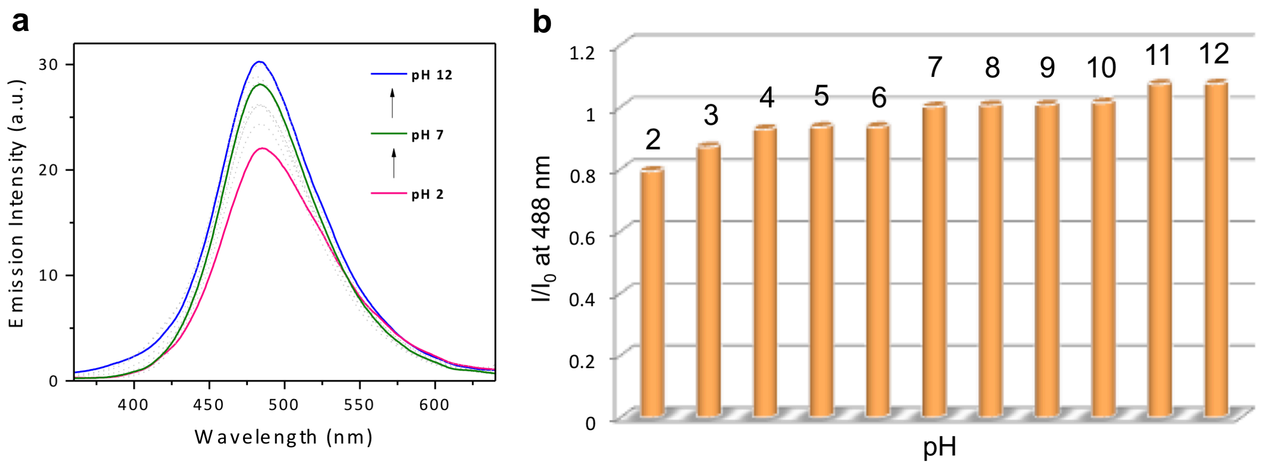

Supplementary Figure S17 | Effect of pH. (a) Fluorescence changes in **PE1** coated over paper substrate on addition water having different pH. (b) Bar diagram representation of fluorescence intensity changes monitored at 488 nm. Fluorescence intensity obtained with application of water at pH 7 is taken as I_0.

Simulated barcode experiment: Solutions of **PE1** and **PE3** (c = 1 x 10^{-3} M) were drop cast on filter papers and dried in a hot air oven to obtain blue emitting filter paper strips. Water was slowly spread towards one of the ends of both the filter papers and the change in emission was recorded with a camera (Nikon – COOLPIX S8200, under ultraviolet 365 nm maintaining the camera at the minimum exposure level of -2). Snapshots of these transitions were collected at different time intervals. A barcode with an encoded message 'Satyameva Jayate' was created from a free barcode generating website (http://online-barcode-generator.net/). This barcode image was then edited in Adobe Photoshop CS2 software and both the white as well as the black portion of the image was removed in separate images. The images were then saved for web in the graphics interchange format (GIF). Then they were grouped with the snapshots obtained from **PE1** and **PE3** film and saved as a single image. These were simultaneously edited in Adobe Photoshop CS2 following the same procedure as stated earlier to remove the black and white portions to create the masks corresponding to the black and white regions of the barcode. These individual masks of **PE1** and **PE3** were then combined with each other to generate a virtual barcode. This procedure was

followed for the time dependent snapshots obtained. A schematic of the entire process is shown in the supporting information (Supplementary Fig. S18). To decode the virtual barcodes in individual cases, a smartphone, installed with a barcode reader application (NeoReader) was used.

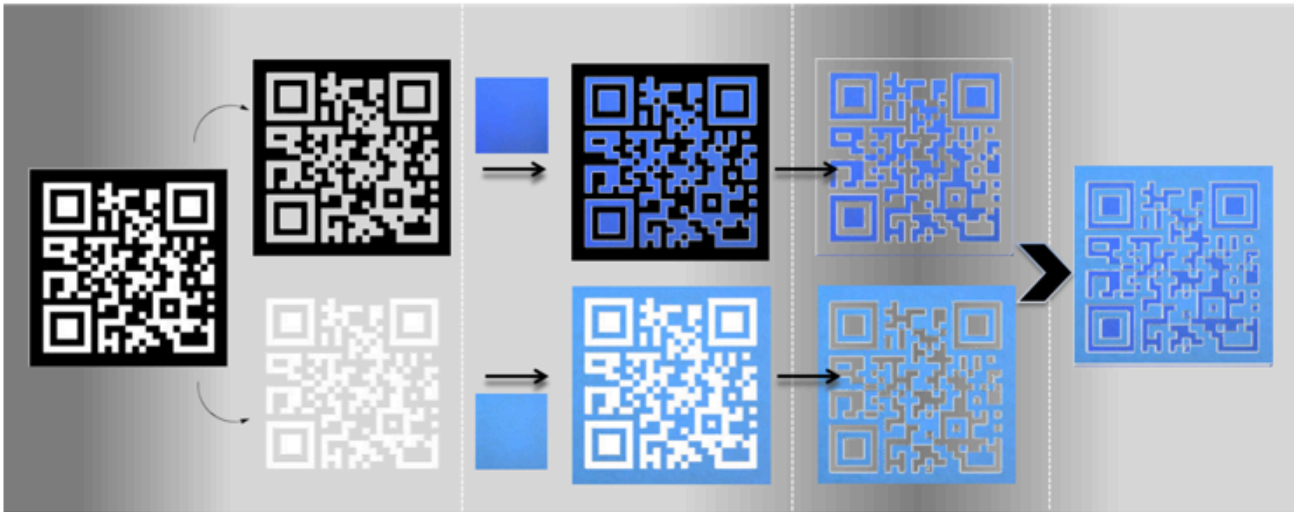

Supplementary Figure S18 | Preparation of masks. Schematic representation of preparation of computer generated masks from **PE1** and **PE3** film photographs, thereby combination of the masks to generate a virtual barcode.

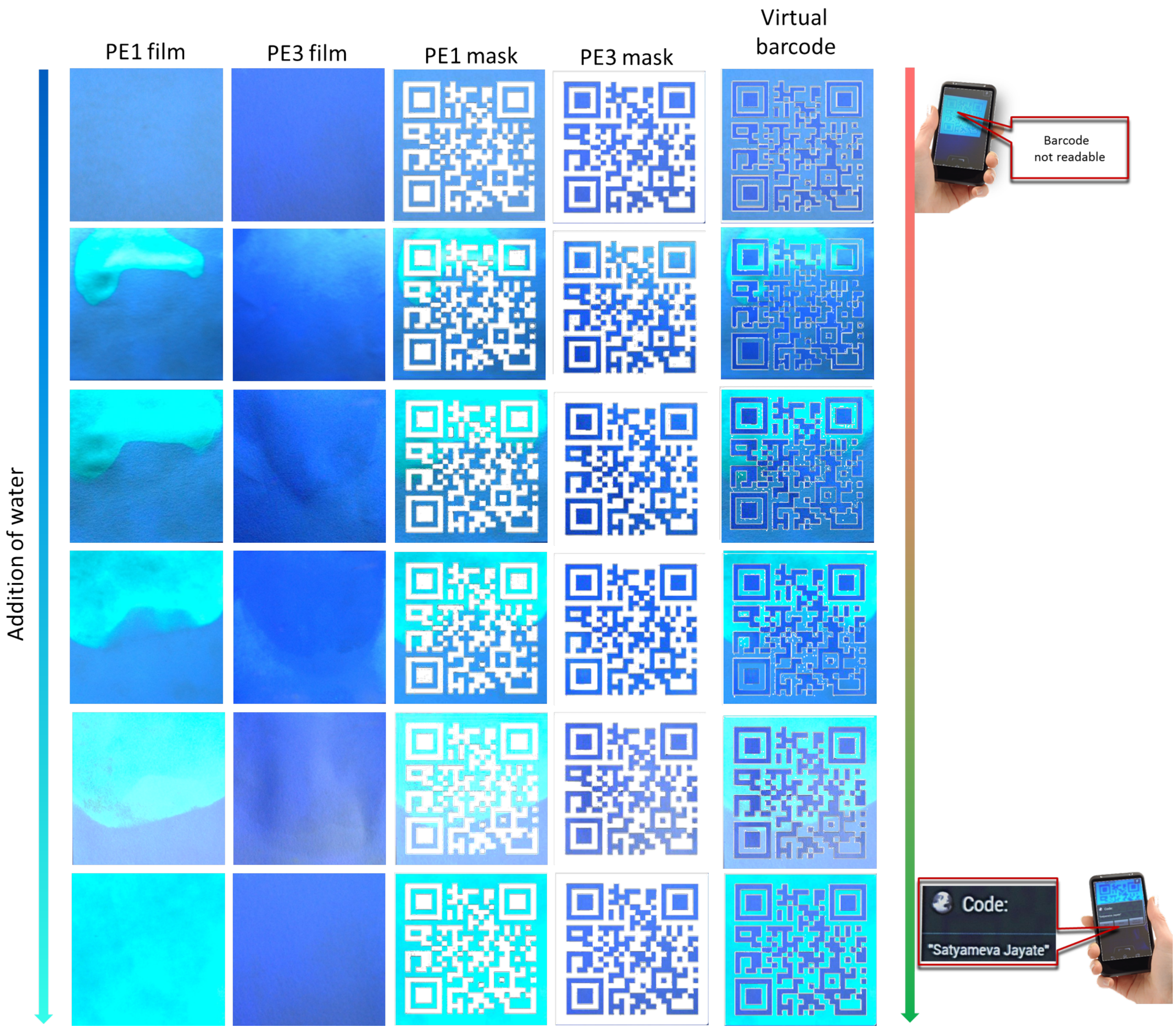

Supplementary Figure S19 | Simulated barcode experiment. Combination of computer generated masks from photographs of **PE1** and **PE3** films to generate a virtual barcode. The barcode remains undetected by a smartphone under normal conditions. On complete wetting by water, initially blue emitting **PE1** film is transformed into a cyan emitting film, thereby generating an optical contrast and the barcode becomes detected.

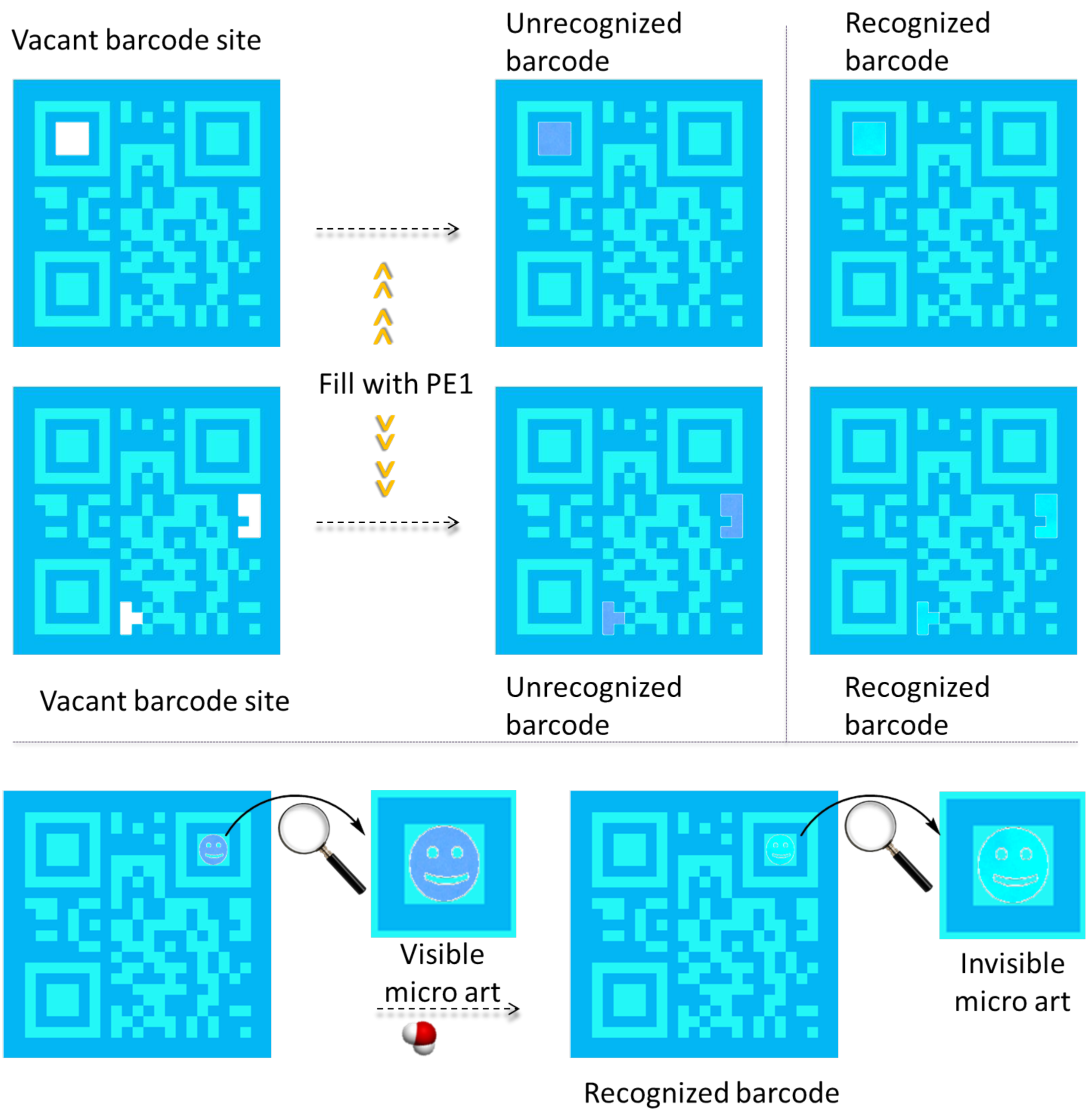

Supplementary Figure S20 | Smart barcode design. Barcodes made up of blue and cyan emitting fluorescent inks. Certain vacant sites (defect sites) can be filled with **PE1,** making the barcode unreadable. A smart phone can only decode the information, only when the **PE1** filled portion is wetted with water. Similar strategy can also be extended to incorporate micro graphics within the barcode.

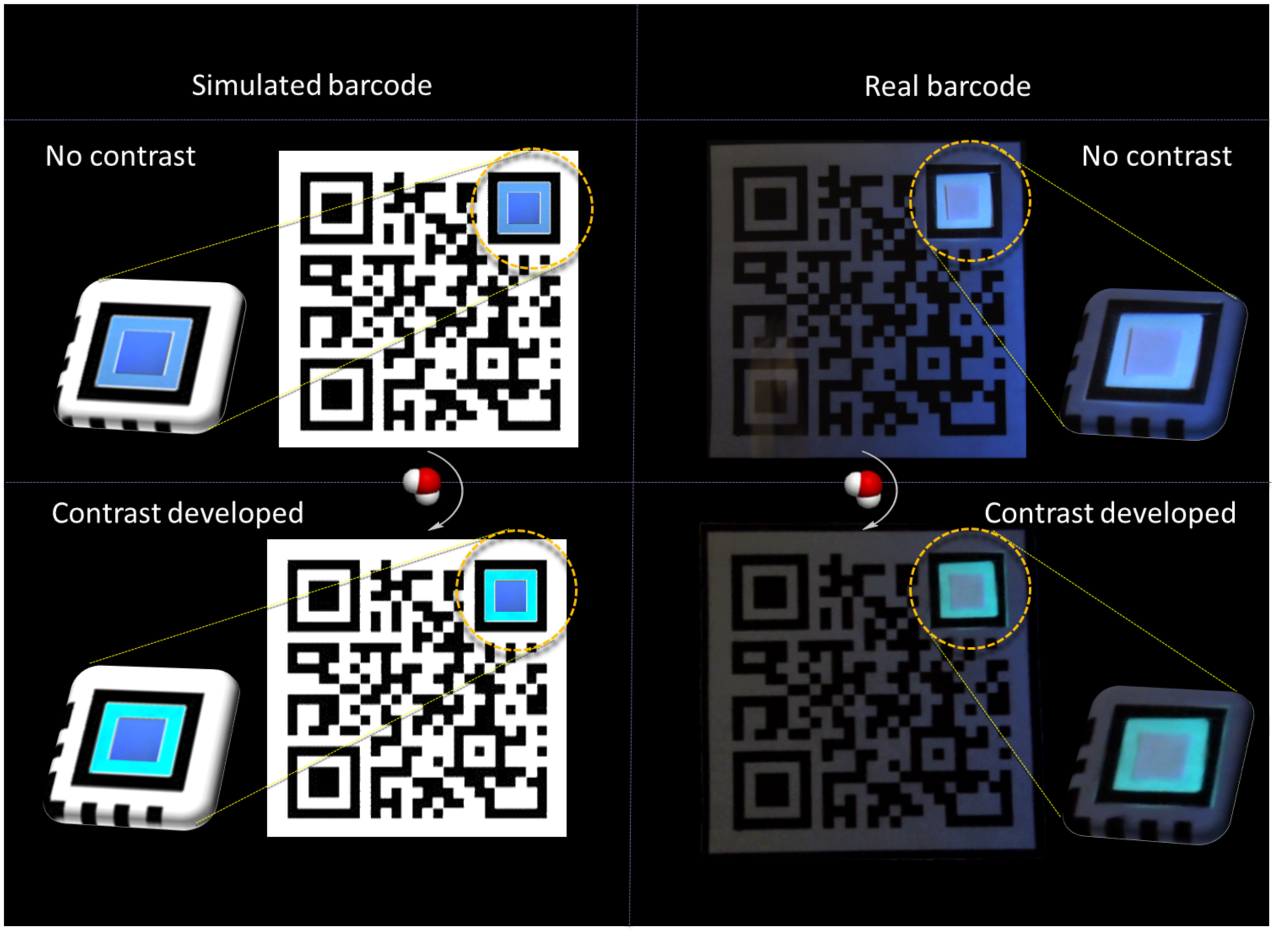

Supplementary Figure S21 | Printed barcode. Barcodes were printed with ordinary ink and the defect sites were filled with **PE1** and **PE3** coated films. Similar optical contrast was developed in the real experimental barcode as observed in the simulated barcode.

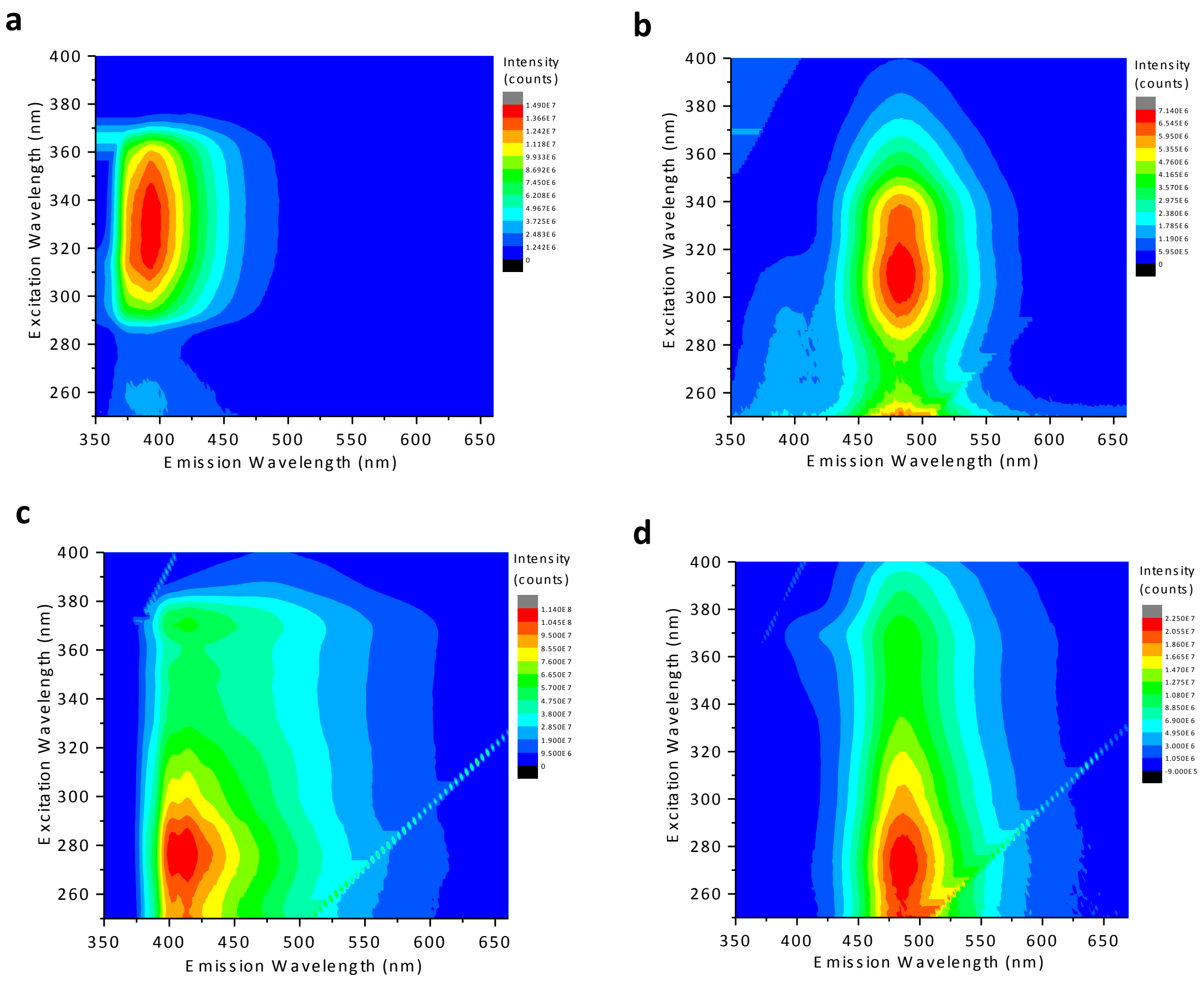

Supplementary Figure S22 | Fluorescence matrix scan experiment. The figures show contour map of the observed fluorescence intensity as a function of the fluorescence emission and excitation wavelength for **PE1**. (a) THF (c = 1 × 10^{-5} M), (b) THF-water (1:9 v/v) mixture (c = 1 × 10^{-5} M), (c) blue emitting film and (d) cyan emitting film.

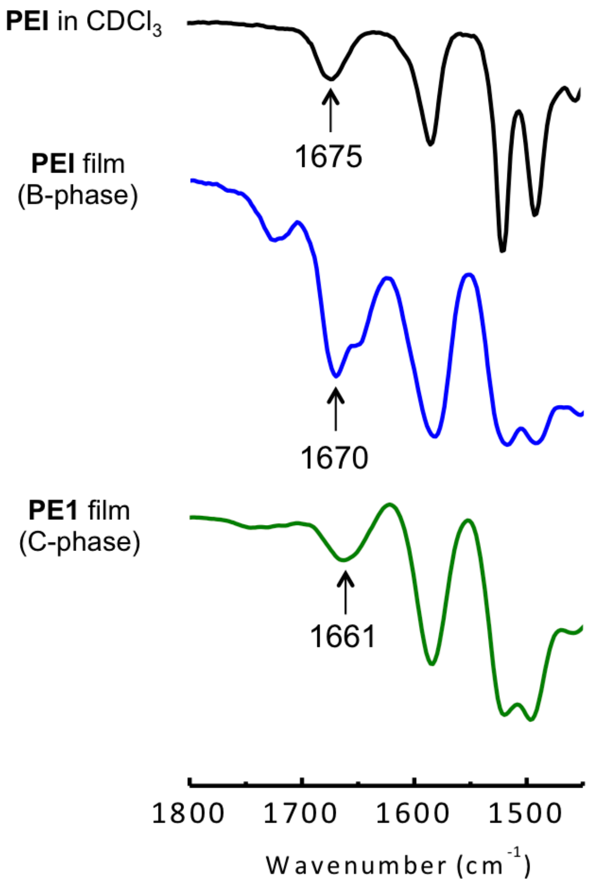

Supplementary Figure S23 | FT-IR Studies. FT-IR spectra of dilute solution of **PE1** in $CDCl_3$ (top), blue emitting film (middle) and cyan emitting film (bottom). Arrows indicate amide carbonyl IR bands.

3. Supplementary Table

Table S1. Quantum yield and fluorescence lifetime characteristics of **PE** derivatives in different states.

Molecule	Solution state	Aggregated state	Blue film	Cyan film
**PE1**	Φ_f = 0.02	Φ_f = 0.14	Φ_f = 0.13	Φ_f = 0.18
	τ = 0.29 ns	τ_1 = 14.95 ns (68%)<br>τ_2 = 5.52 ns (29%)<br>τ_3 = 1.10 ns (3%)	τ_1 = 1.04 ns (72%)<br>τ_2 = 5.29 ns (18%)<br>τ_3 = 2.92 ns (10%)	τ_1 = 15.25 ns (47%)<br>τ_2 = 5.84 ns (44%)<br>τ_3 = 1.68 ns (9%)
**PE2**	Φ_f = 0.02	Φ_f = 0.18	---	Φ_f = 0.22
	τ = 0.78 ns	τ_1 = 23.35 ns (60%)<br>τ_2 = 8.55 ns (34%)<br>τ_3 = 1.68 ns (6%)	---	τ_1 = 21.54 ns (52%)<br>τ_2 = 8.23 ns (41%)<br>τ_3 = 1.44 ns (7%)
**PE3**	Φ_f = 0.02	Φ_f = 0.34	Φ_f = 0.32	---
	τ = 0.19 ns	τ_1 = 1.57 ns (52%)<br>τ_2 = 0.14 ns (44%)<br>τ_3 = 4.74 ns (4%)	τ_1 = 0.91 ns (50%)<br>τ_2 = 0.51 ns (47%)<br>τ_3 = 3.41 ns (3%)	---

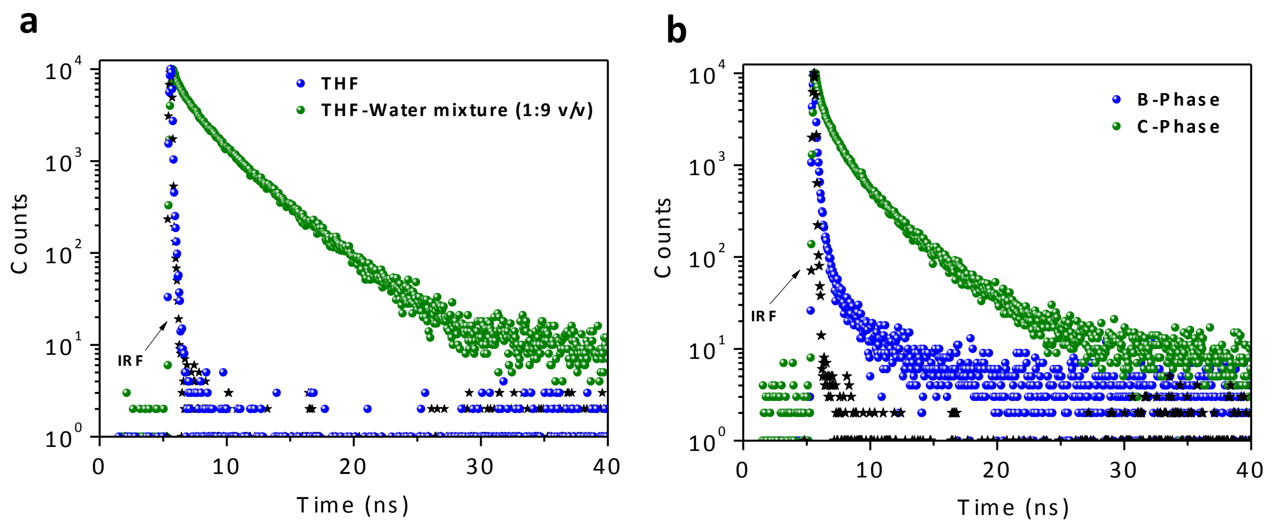

Supplementary Figure S24 | Fluorescence lifetime studies. Emission decay profiles of **PE1** in (a) THF (blue) and THF-Water mixture (1:9 v/v) (green), (b) blue-emitting film (blue) and cyan-emitting film (green). Fluorescence decay profiles were recorded by excitation at 375 nm, emission monitored at the emission maximum.

4. Fluorescence Quantum Yield Measurements

Fluorescence quantum yield (Φ_s) of **PE** derivatives was determined using quinine sulfate (Φ_r = 0.546 in 0.1 N H_2SO_4) as the reference standard. The experiments were done using optically matching solutions and the quantum yield was calculated using equation 1[S1],

$$\Phi_s = \Phi_r \, (A_r F_s / A_s F_r) \, (\eta_s^2/\eta_r^2) \qquad \text{-------- (1)}$$

where, A_s and A_r are the absorbance of the sample and reference solutions respectively at the same excitation wavelength, F_s and F_r are the corresponding relative integrated fluorescence intensities and η is the refractive index of the solvent.

Fluorescence quantum yield in the film state were measured using a calibrated integrating sphere attached to a SPEX Fluorolog spectrofluorimeter. A Xe arc lamp was used to excite the

sample placed in the sphere with λ = 340 nm as the excitation wavelength. The absolute fluorescence quantum yield was calculated based on the de Mello method[32,S3] by using Equation (2):

$$\Phi_{PL} = [E_i(\lambda) - (1-A)\, E_0(\lambda)] \,/\, L_e(\lambda)A \qquad \text{---------(2)}$$

For equation (2):

$$A = [L_0(\lambda) - L_i(\lambda)] \,/\, L_0(\lambda) \qquad \text{----------(3)}$$

where $E_i(\lambda)$ and $E_0(\lambda)$ are the integrated luminescence as a result of the direct excitation of sample and secondary excitation, respectively; A is the absorbance of the sample calculated using Equation (3); $L_i(\lambda)$ is the integrated excitation when the sample is directly excited; $L_0(\lambda)$ is the integrated excitation when the excitation light first hits the sphere and reflects into the sample; and $L_e(\lambda)$ is the integrated excitation profile for an empty sphere.

5. Supporting References

S1. Lakowicz, J. R. Principles of fluorescence spectroscopy, 3rd ed., Springer, New York (2006).

S2. de Mello, J. C., Wittmann, H. F. & Friend, R. T. An Improved Experimental Determination of External Photoluminescence Quantum Efficiency. *Adv. Mater.* **9,** 230-232 (1997).

S2. Palsson, L.-O. & Monkman, A. P. Measurements of solid-state photoluminescence quantum yields of films using a fuorimeter. *Adv. Mater.* **14,** 757-758 (2002).
